# Machine learning identifies signatures of macrophage reactivity and tolerance that predict disease outcomes

**DOI:** 10.1016/j.ebiom.2023.104719

**Published:** 2023-07-27

**Authors:** Pradipta Ghosh, Saptarshi Sinha, Gajanan D. Katkar, Daniella Vo, Sahar Taheri, Dharanidhar Dang, Soumita Das, Debashis Sahoo

**Affiliations:** a Department of Cellular and Molecular Medicine, University of California San Diego, USA; b Department of Medicine, University of California San Diego, USA; c Moores Cancer Center, University of California San Diego, USA; d Department of Pediatrics, University of California San Diego, USA; e Department of Pathology, University of California San Diego, USA; f Department of Computer Science and Engineering, Jacob’s School of Engineering, University of California San Diego, USA

**Keywords:** Artificial intelligence, Boolean equivalent clusters, Macrophage, Reactive, Tolerant, Innate immune response, Outcome prediction

## Abstract

**Background:**

Single-cell transcriptomic studies have greatly improved organ-specific insights into macrophage polarization states are essential for the initiation and resolution of inflammation in all tissues; however, such insights are yet to translate into therapies that can predictably alter macrophage fate.

**Method:**

Using machine learning algorithms on human macrophages, here we reveal the continuum of polarization states that is shared across diverse contexts. A path, comprised of 338 genes accurately identified both physiologic and pathologic spectra of “*reactivity*” and “*tolerance*”, and remained relevant across tissues, organs, species, and immune cells (>12,500 diverse datasets).

**Findings:**

This 338-gene signature identified macrophage polarization states at single-cell resolution, in physiology and across diverse human diseases, and in murine pre-clinical disease models. The signature consistently outperformed conventional signatures in the degree of transcriptome-proteome overlap, and in detecting disease states; it also prognosticated outcomes across diverse acute and chronic diseases, e.g., sepsis, liver fibrosis, aging, and cancers. Crowd-sourced genetic and pharmacologic studies confirmed that model-rationalized interventions trigger predictable macrophage fates.

**Interpretation:**

These findings provide a formal and universally relevant definition of macrophage states and a predictive framework (http://hegemon.ucsd.edu/SMaRT) for the scientific community to develop macrophage-targeted precision diagnostics and therapeutics.

**Funding:**

This work was supported by the National Institutes for Health (10.13039/100000002NIH) grant R01-AI155696 (to P.G, D.S and S.D). Other sources of support include: R01-GM138385 (to D.S), R01-AI141630 (to P.G), R01-DK107585 (to S.D), and UG3TR003355 (to D.S, S.D, and P.G). D.S was also supported by two Padres Pedal the Cause awards (Padres Pedal the Cause/RADY #PTC2017 and San Diego 10.13039/100000054NCI Cancer Centers Council (C3) #PTC2017). S.S, G.D.K, and D.D were supported through 10.13039/100002570The American Association of Immunologists (AAI) Intersect Fellowship Program for Computational Scientists and Immunologists. We also acknowledge support from the Padres Pedal the Cause #PTC2021 and the Torey Coast Foundation, La Jolla (P.G and D.S). D.S, P.G, and S.D were also supported by the 10.13039/100007028Leona M. and Harry B. Helmsley Charitable Trust.

**Research in context**

**Evidence before this study**

The concept of macrophage polarization is well established in immunology and has been widely studied. There are multiple biomarkers that have been used to distinguish between reactive (M1) and tolerant (M2) macrophages, such as the expression of specific surface receptors, cytokines, and metabolic enzymes. The use of biomarkers for macrophage polarization is not always straightforward, as the phenotype of macrophages can be influenced by multiple factors and can vary between different tissues. There is no universal biomarker of macrophage polarization that can be used across all tissues and conditions.

**Added value of this study**

This work identifies a Signature of Macrophage Reactivity and Tolerance (SMaRT) that is surprisingly conserved in many tissues and conditions. A set of 338-genes derived from Boolean Implication Network model of macrophages identified macrophage polarization states in single cell, in diverse physiology, tissue and disease context. The signature was strongly associated with outcome in several diseases. Further, genetic, and pharmacologic manipulations of several SMaRT genes were found to modulate macrophage polarization exactly as predicted by the model.

**Implications of all the available evidence**

The SMaRT signatures provide a quantitative and qualitative framework for assessing macrophage polarization across diverse tissues and conditions. The genes identified here reveal several hitherto unforeseen players of macrophage polarizations and potentially high-value targets to manipulate the same.

## Introduction

Macrophages are complex; as sentinel cells of the innate immune system, they are found in various organs and their dysregulated activation can directly impact organ functions and the outcome of all diseases.^1^^,^^2^ Macrophages were initially classified as M1 (the classically activated macrophages) and M2 (the alternatively activated macrophages) based on their functions at the extremes of polarization states.^3^ However, the current M1 and/or M2 signatures fail to describe the diverse, polyfunctional and plastic cells, and the myriad of continuum states that they adopt in the tissue at steady-state and during disease.^4^, ^5^, ^6^, ^7^ To cope with this degree of diversity and plasticity, several definitions of macrophage subtypes have emerged, each representing specialized contexts, e.g., TAMs, tumour-associated macrophages^8^; LAMs, lipid-associated macrophages in atherosclerosis^9^; DAMs, disease-associated microglia in neurodegenerative disorders^10^; SAMs, scar-associated macrophages in liver fibrosis.^11^, ^12^, ^13^ These definitions were geared to identify divergent markers, spatial localization, origin, and functional pathways associated with macrophages during disease; however, they fall short in predictive or prognostic abilities.

**Fig. 1:**
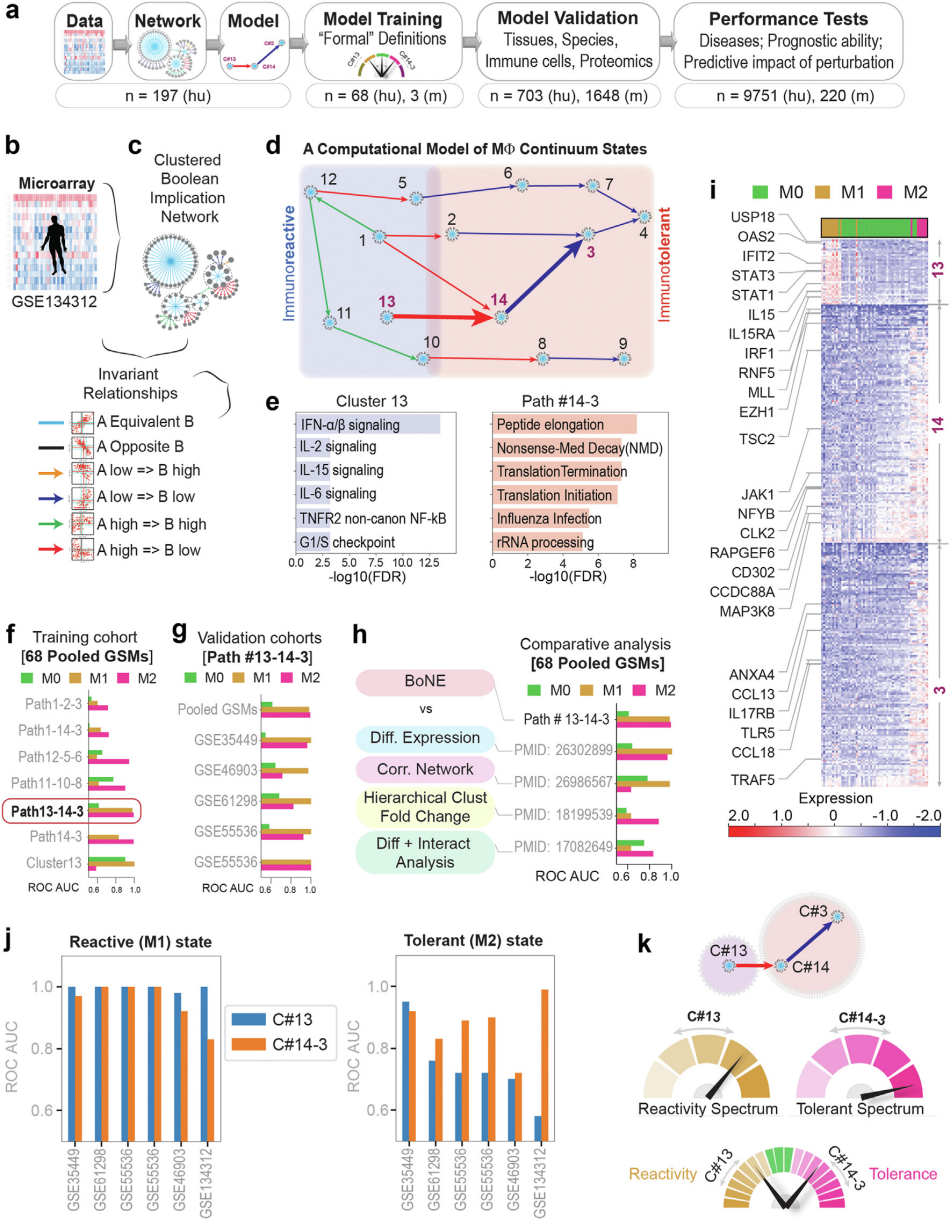
***BoNE*-assisted formulation of formal definitions of macrophage polarization. a)** Overview of workflow and approach used in this work. **b and c)** A pooled dataset of diverse human transcriptomes (**b**; n = 197) was used to build a Boolean implication network (**c**-*top*) and visualized as gene clusters (nodes, comprised of genes that are equivalent to each other) that are interconnected based on one of the six overwhelming Boolean implication relationship between the clusters (directed edges; **c**-*bottom*). **d)** Display of the major Boolean paths within the network prioritized based on the cluster size. Annotations of “immunoreactive” and “immunotolerant” ends of the spectrum are based on the expression profile of the gene clusters in 68 samples within the pooled dataset that were stimulated in vitro as M1 and M2, respectively. **e)** Reactome pathway analysis of each cluster along the top continuum paths was performed to identify the enriched pathways (for other clusters see http://hegemon.ucsd.edu/SMaRT/). **f and g)** Training (**f**) was performed on the 68 pooled samples using machine-learning approaches; the best-performing Boolean path, #13-14-3 was then validated (**g**) in multiple independent human macrophage datasets. For a list of datasets used see Supplementary Table S1. The performance was measured by computing ROC AUC for a logistic regression model. **h)** Comparative analysis of performance of the *BoNE*-derived *versus* other traditional approaches in segregating M0/M1/M2 polarization states. **i)** Heatmap displaying the pattern of gene expression in C#13, 14 and 3. Selective genes are labelled. **j)** Validation studies assessing the ability of the genes in either C#13 alone or C#14-3 alone to classify M0/M1/M2 polarization states in multiple human macrophage datasets. **k)***Top*: Schematic summarizing the model-derived formal definitions of macrophage polarization states based on the levels of expression of genes in C#13 (hypo to hyper- “reactivity” spectrum) and those in C#14 + 3 (hypo to hyper- “tolerant” spectrum). *Bottom*: A composite score of the entire range of physiologic and pathologic response can be assessed via the *BoNE*-derived path #13 → 14 → 3.

We sought to create and validate a comprehensive model of macrophage processes for defining, tracking, and even predicting macrophage fate after perturbation (see Fig. 1a and Supplementary Fig. S1A for workflow outline). We hypothesized that such a model might inspire *formal* definitions for macrophage polarization states that are reflective of fundamental processes and maintain relevance across tissues, organs, diseases and species. In addition, it may also rationalize diagnostics and therapeutics to detect and reset, respectively, deranged macrophage states in disease. We show that such formal definition(s) of macrophage states is not only possible, but also provide evidence for their usefulness in single cell data analysis, in prediction and prognostication.

## Methods

**Table:** Key resource table

Reagent or resource	Source	Identifier
**Deposited data**		
Pooled human macrophage array *Ccdc88a* KO peritoneal macrophages	NCBI GEO (The National Center for Biotechnology Information- Gene expression omnibus)	GSE134312 GSE203423
Proteomics dataset, reanalysed from PMID: 34731634	MassIVE repository	MSV000084672
**Experimental models**: Organisms/strains		
*Ccdc88a fl/fl LysMCre/- mice*	PMID: 33055214	
**Software and algorithms**		
Numpy	Python	https://numpy.org
Scipy	Python	https://scipy.org
Seaborn	Python	https://seaborn.pydata.org
Matplotlib	Python	https://matplotlib.org
Hierarchical exploration of gene expression microarrays online (Hegemon)	HTML, JavaScript, Python, PHP	https://github.com/sahoo00/Hegemon
Boolean network explorer (*BoNE*)	Python	https://github.com/sahoo00/BoNE
Other		
Interactive website	This paper	http://hegemon.ucsd.edu/SMaRT/

### Detailed methods

#### Data collection and annotation

Publicly available microarray and RNASeq databases were downloaded from the National Center for Biotechnology Information (NCBI) Gene Expression Omnibus (GEO) website.^14^, ^15^, ^16^ Gene expression summarization was performed by normalizing Affymetrix platforms by RMA (Robust Multichip Average)^17^^,^^18^ and RNASeq platforms by computing TPM (Transcripts Per Millions)^19^ values whenever normalized data were not available in GEO. We used log2(TPM) if TPM > 1 and (TPM—1) if TPM < 1 as the final gene expression value for analyses. We also used log2(TPM + 1) in some datasets. We also used publicly data normalized using RPKM,^20^ FPKM,^21^^,^^22^ TPM,^23^^,^^24^ and CPM.^25^^,^^26^ In the context of Affymetrix microarray data we believe that RMA works better than MAS 5.0.^27^

##### Macrophage datasets used for network analysis

Previously published pooled macrophage dataset from GEO (GSE134312, n = 197) assayed on the Human U133 Plus 2.0 (GPL570), Human U133A 2.0 (GPL571) and Human U133A (GPL96) platforms were used to perform macrophage network analysis. This dataset was manually annotated with M0, M1 or M2 phenotypes. Accession numbers for the M0, M1, and M2 phenotypes are presented in Supplementary Table S1. Five validation datasets are used to test the macrophage gene signature: GSE35449 (7 M0, 7 M1, 7 M2), GSE46903 (64 M0, 29 M1, 40 M2), GSE61298 (6 M0, 6 M1, 6 M2), GSE55536 human peripheral blood mononuclear cell-derived macrophage (6 M0, 6 M1, 6 M2), GSE55536 iPSC derived macrophages (3 M0, 3 M1, 3 M2). See Supplementary Information 1 for all datasets analysed in this work.

#### Computational approaches

##### StepMiner analysis

StepMiner is a computational tool that identifies step-wise transitions in a time-series data.^28^ StepMiner performs an adaptive regression scheme to identify the best possible step up or down based on sum-of-square errors. The steps are placed between time points at the sharpest change between low expression and high expression levels, which gives insight into the timing of the gene expression-switching event. To fit a step function, the algorithm evaluates all possible step positions, and for each position, it computes the average of the values on both sides of the step for the constant segments. An adaptive regression scheme is used that chooses the step positions that minimize the square error with the fitted data. Finally, a regression test statistic is computed as follows:

$$F\;stat=\frac{\sum_{i=1}^{n}{\left({\hat{X}}_{i}\;-\;\bar{X}\right)}^{2}/\left(m\;-\;1\right)}{\sum_{i=1}^{n}{\left(X_{i}\;-\;{\hat{X}}_{i}\right)}^{2}/\left(n\;-\;m\right)}$$

Where $X_{i}$ for i=1 to n are the values, $\hat{X_{i}}$ for i=1 to n are fitted values. m is the degrees of freedom used for the adaptive regression analysis. $\bar{X}$ is the average of all the values: $\bar{X}=\frac{1}{n}*\;\sum_{j=1}^{n}X_{j}$. For a step position at k, the fitted values $\hat{X_{l}}$ are computed by using $\frac{1}{k}*\;\sum_{j=1}^{n}X_{j}$ for i=1 to k and $\frac{1}{\left(n\;-\;k\right)}*\;\sum_{j=k+1}^{n}X_{j}$ for i=k+1 to n.

##### Boolean analysis

**Fig. 2:**
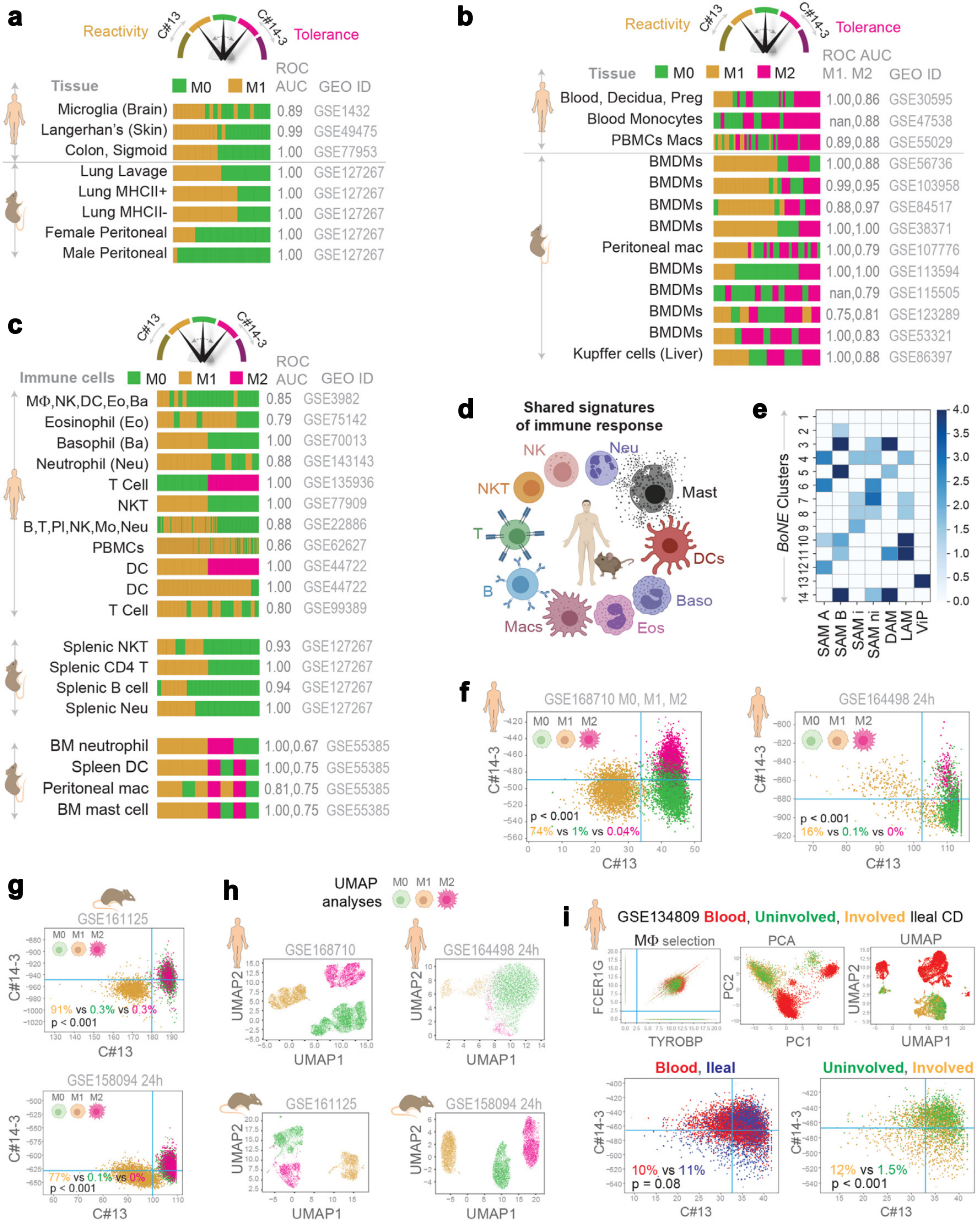
**Definitions of “reactivity” and “tolerance” are conserved across tissues, organs, species, and diverse immune cell types. a and b)** Validation studies assessing the ability of *SMaRT* genes to classify diverse tissue-resident macrophage datasets from both humans and mice. Performance is measured by computing ROC-AUC. Barplots show the ranking order of different sample types based on the composite scores of C#13 and path #14-3. **c and d)** Validation studies (**c**) assessing the ability of *SMaRT* genes to classify active vs inactive states of diverse immune cell types in both humans and mice. The schematic (**d**) summarizes findings in c. **e)** Published disease-associated macrophage gene signatures (see Supplemental Information 2) are analysed for significant overlaps with various gene clusters in the Boolean map of macrophage processes. Results are displayed as heatmaps of -Log10(*p)* values as determined by a hypergeometric test. **f and g)** Scatterplots of the composite score of C#13 and path #14-3 in human (**f**, GSE168710, GSE164498 24 h) and mouse (**g**, GSE161125, GSE158094 24 h) single cell RNASeq datasets with well defined macrophage polarization states (M0, M1, M2). Blue lines correspond to the StepMiner thresholds. Percentages of different cell types are reported in the bottom-left quadrant. Pvalue is computed by two tailed two proportions z-test for M1 vs M0. **h)** Traditional UMAP analysis of the single cell RNASeq datasets. **i)** PCA, UMAP and BoNE analysis of single cell RNASeq dataset GSE134809 that includes blood and ileal biopsy (uninvolved and involved) samples from Crohn’s disease (CD) patients. Macrophages were selected as the top right corner by using thresholds (2.5, blue lines) on TYROBP and FCER1G. Blue lines correspond to the StepMiner thresholds in the scatterplot between C#13 and C#14-3 (bottom plots). Bottom-left quadrant is evaluated for enrichment of cell types across tissue (blood vs ileal) and disease states (uninvolved vs involved CD). Percentages of different cell types are reported in the bottom-left quadrant. P value is computed by two tailed two proportions z-test.

**Boolean logic** is a simple mathematic relationship of two values, i.e., high/low, 1/0, or positive/negative. The Boolean analysis of gene expression data requires the conversion of expression levels into two possible values. The ***StepMiner*** algorithm is reused to perform Boolean analysis of gene expression data.^29^ **The Boolean analysis** is a statistical approach which creates binary logical inferences that explain the relationships between phenomena. Boolean analysis is performed to determine the relationship between the expression levels of pairs of genes. The ***StepMiner*** algorithm is applied to gene expression levels to convert them into Boolean values (high and low). In this algorithm, first the expression values are sorted from low to high and a rising step function is fitted to the series to identify the threshold. Middle of the step is used as the StepMiner threshold. This threshold is used to convert gene expression values into Boolean values. A noise margin of 2-fold change is applied around the threshold to determine intermediate values, and these values are ignored during Boolean analysis. In a scatter plot, there are four possible quadrants based on Boolean values: (low, low), (low, high), (high, low), (high, high). A Boolean implication relationship is observed if any one of the four possible quadrants or two diagonally opposite quadrants are sparsely populated. Based on this rule, there are six kinds of Boolean implication relationships. Two of them are symmetric: equivalent (corresponding to the positively correlated genes), opposite (corresponding to the highly negatively correlated genes). Four of the Boolean relationships are asymmetric, and each corresponds to one sparse quadrant: (low => low), (high => low), (low => high), (high => high). BooleanNet statistics (Fig. 2a) is used to assess the sparsity of a quadrant and the significance of the Boolean implication relationships.^29^^,^^30^ Given a pair of genes A and B, four quadrants are identified by using the StepMiner thresholds on A and B by ignoring the Intermediate values defined by the noise margin of 2 fold change ( ± 0.5 around StepMiner threshold). Number of samples in each quadrant are defined as a_00_, a_01_, a_10_, and a_11_ (Fig. 1a) which is different from X in the previous equation of F stat. Total number of samples where gene expression values for A and B are low is computed using the following equations.

$$nA_{low}=\left(a_{00}+a_{01}\right),nB_{low}=\left(a_{00}+a_{10}\right)\text{,}$$

Total number of samples considered is computed using following equation.

$$total=a_{00}\;+\;a_{01}\;+\;a_{10}\;+a_{11}$$

Expected number of samples in each quadrant is computed by assuming independence between A and B. For example, expected number of samples in the bottom left quadrant e_00_ = $\hat{n}$ is computed as probability of A low ((a_00_ + a_01_)/total) multiplied by probability of B low ((a_00_ + a_10_)/total) multiplied by total number of samples. Following equation is used to compute the expected number of samples.

$$n=a_{ij},\hat{n}=\left({nA}_{low}/total*\;{nB}_{low}/total\right)*total$$

To check whether a quadrant is sparse, a statistical test for (e_00_ > a_00_) or ($\hat{n}>n)$ is performed by computing S_00_ and p_00_ using following equations. A quadrant is considered sparse if S_00_ is high ($\hat{n}>n)$ and p_00_ is small.

$$S_{ij}=\frac{\hat{n}\;-\;n}{\sqrt{\hat{n}}}$$

$$p_{00}=\frac{1}{2}\left(\frac{a_{00}}{\left(a_{00}+a_{01}\right)}+\frac{a_{00}}{\left(a_{00}+a_{10}\right)}\right)$$

A suitable threshold is chosen for S_00_ > sThr and p_00_ < pThr to check sparse quadrant. A Boolean implication relationship is identified when a sparse quadrant is discovered using following equation.

$$Boolean\;Implication=\left(S_{ij}>\text{sThr},p_{ij}<\text{pThr}\right)$$

A relationship is called Boolean equivalent if top-left and bottom-right quadrants are sparse.

$$Equivalent=(S_{01}>sThr,P_{01}<pThr,S_{10}>sThr,P_{10}<pThr)$$

Boolean opposite relationships have sparse top-right (a_11_) and bottom-left (a_00_) quadrants.

$$Opposite=(S_{00}>sThr,P_{00}<pThr,S_{11}>sThr,P_{11}<pThr)$$

Boolean equivalent and opposite are symmetric relationship because the relationship from A to B is same as from B to A. Asymmetric relationship forms when there is only one quadrant sparse (A low => B low: top-left; A low => B high: bottom-left; A high => B high: bottom-right; A high => B low: top-right). These relationships are asymmetric because the relationship from A to B is different from B to A. For example, A low => B low and B low => A low are two different relationships.

A low => B high is discovered if the bottom-left ($a_{00}$) quadrant is sparse and this relationship satisfies following conditions.

$$A\;low=>\;B\;high=(S_{00}>sThr,P_{00}<pThr)$$

Similarly, A low => B low is identified if the top-left (a_01_) quadrant is sparse.

$$A\;low=>\;B\;low=(S_{01}>sThr,P_{01}<pThr)$$

A high => B high Boolean implication is established if the bottom-right (a_10_) quadrant is sparse as described below.

$$A\;high=>\;B\;high=(S_{10}>sThr,P_{10}<pThr)$$

Boolean implication A high => B low is found if the top-right (a_11_) quadrant is sparse using following equation.

$$A\;high=>\;B\;low=(S_{11}>sThr,P_{11}<pThr)$$

For each quadrant a statistic S_ij_ and an error rate p_ij_ is computed. S_ij_ > sThr and p_ij_ < pThr are the thresholds used on the BooleanNet statistics to identify Boolean implication relationships.

Boolean analyses in the test dataset GSE134312 uses a threshold of sThr = 3 and pThr = 0.1. These thresholds are exactly same as the previously used thresholds sThr = 3 and pThr = 0.1 for BooleanNet.^27^^,^^29^^,^^31^ False discovery rate is computed for these thresholds (FDR <0.000001) by using randomly permuting gene expression data in GSE134312.

##### Boolean network explorer (BoNE)

Boolean network explorer (*BoNE*) provides an integrated platform for the construction, visualization and querying of a network of progressive changes underlying a disease or a biological process in three steps (Supplementary Fig. S1A): First, the expression levels of all genes in these datasets were converted to binary values (high or low) using the StepMiner algorithm. Second, gene expression relationships between pairs of genes were classified into one-of-six possible Boolean Implication Relationships (BIRs), two symmetric and four asymmetric, and expressed as Boolean implication statements. This offers a distinct advantage from conventional computational methods (Bayesian, Differential, etc.) that rely exclusively on symmetric linear relationships in networks. The other advantage of using BIRs is that they are robust to the noise of sample heterogeneity (i.e., healthy, diseased, genotypic, phenotypic, ethnic, interventions, disease severity) and every sample follows the same mathematical equation, and hence is likely to be reproducible in independent validation datasets. Third, genes with similar expression architectures, determined by sharing at least half of the equivalences among gene pairs, were grouped into clusters and organized into a network by determining the overwhelming Boolean relationships observed between any two clusters. In the resultant Boolean implication network, clusters of genes are the nodes, and the BIR between the clusters are the directed edges; *BoNE* enables their discovery in an unsupervised way while remaining agnostic to the sample type.

##### Statistical analyses

Gene signature is used to classify sample categories and the performance of the multi-class classification is measured by ROC-AUC (Receiver Operating Characteristics Area Under The Curve) values. A color-coded bar plot is combined with a density or violin + swarm plot to visualize the gene signature-based classification. All statistical tests were performed using R version 3.2.3 (2015-12-10). Standard t-tests were performed using python scipy.stats.ttest_ind package (version 0.19.0) with Welch’s Two Sample t-test (unpaired, unequal variance (equal_var = False), and unequal sample size) parameters. Multiple hypothesis corrections were performed by adjusting *p* values with statsmodels.stats.multitest.multipletests (fdr_bh: Benjamini/Hochberg principles). The results were independently validated with R statistical software (R version 3.6.1; 2019-07-05). Pathway analysis of gene lists were carried out via the Reactome database and algorithm.^32^ Reactome identifies signalling and metabolic molecules and organizes their relations into biological pathways and processes. Kaplan–Meier analysis is performed using lifelines python package version 0.14.6.

##### Boolean implication network construction

A Boolean implication network (BIN) is created by identifying all significant pairwise Boolean implication relationships (BIRs) for GSE134312 datasets (Supplementary Fig. S1B). The Boolean implication network contains the six possible Boolean relationships between genes in the form of a directed graph with nodes as genes and edges as the Boolean relationship between the genes. The nodes in the BIN are genes and the edges correspond to BIRs. Equivalent and Opposite relationships are denoted by undirected edges and the other four types (low => low; high => low; low => high; high => high) of BIRs are denoted by having a directed edge between them. The network of equivalences seems to follow a scale-free trend; however, other asymmetric relations in the network do not follow scale-free properties. BIR is strong and robust when the sample sizes are usually more than 200. However, it is also possible to build BIN for smaller dataset such as the selected macrophage GSE134312 dataset (n = 197). The macrophage dataset GSE134312 was prepared for Boolean analysis by filtering genes that had a reasonable dynamic range of expression values. When the dynamic range of expression values was small, it was difficult to distinguish if the values were all low or all high or there were some high and some low values. Thus, it was determined to be best to ignore them during Boolean analysis. The filtering step was performed by analyzing the fraction of high and low values identified by the StepMiner algorithm.^28^ Any probe set or genes which contained less than 5% of high or low values were dropped from the analysis.

##### Clustered Boolean Implication network

Clustering was performed in the Boolean implication network to dramatically reduce the complexity of the network (Supplementary Fig. S1C). A clustered Boolean implication network (CBIN) was created by clustering nodes in the original BIN by following the equivalent BIRs. One approach is to build connected components in a undirected graph of Boolean equivalences. However, because of noise the connected components become internally inconsistent e.g., two genes opposite to each other becomes part of the same connected component. In order to avoid such situation, we need to break the component by removing the weak links. To identify the weakest links, we first computed a minimum spanning tree for the graph and computed Jaccard similarity coefficient for every edge in this tree. Ideally if two members are part of the same cluster they should share as many connections as possible. If they share less than half of their total individual connections (Jaccard similarity coefficient less than 0.5) the edges are dropped from further analysis. Thus, many weak equivalences were dropped using the above algorithm leaving the clusters internally consistent. We removed all edges that have Jaccard similarity coefficient less than 0.5 and built the connected components with the rest. The connected components were used to cluster the BIN which is converted to the nodes of the CBIN. Increasing the Jaccard similarity cut-off will result in more compact and correlated clusters in CBIN. The distribution of cluster sizes was plotted in a log–log scale to observe the characteristic of the Boolean network (Supplementary Fig. S1D). To ensure that the cluster sizes exhibit scale-free properties, the Jaccard similarity cut-off is modified such that they are evenly distributed along a straight line on a log–log plot (Supplementary Fig. S1D). A new graph was built that connected the individual clusters to each other using Boolean relationships. Genes in each cluster is ranked based on the number of equivalences within the cluster. Link between two clusters (A, B) was established by using the top representative node from A that was connected to most of the member of A and sampling 6 nodes from cluster B and identifying the overwhelming majority of BIRs (Supplementary Fig. S1C) between the nodes from each cluster. The 6 nodes include the top representative gene (first rank), the gene next to top (second rank), middle (floor (n/2)^th^ rank where n is the cluster size), gene next to middle (floor (n/2)—1 rank), middle from top half (floor (n/4)^th^ ranked gene), and middle from the top 1/4th (floor (n/8)^th^ ranked gene) representative nodes from cluster B if size of the cluster is greater than 10. If size of the cluster is between 2 and 10, top two and middle one is picked to test the relationship with cluster A. If the size of the cluster is 1, then it is used to test the relationship with cluster A. Testing multiple nodes provides the most common type of relationships found between cluster A and B. We suggest referring the codebase released for additional details.

A CBIN was created using the selected GSE134312 datasets. Each cluster was associated with reactive or tolerant macrophage samples based on where these gene clusters were highly expressed. The edges between the clusters represented the Boolean relationships that are color-coded as follows: orange for low => high, dark blue for low => low, green for high => high, red for high => low, light blue for equivalent and black for opposite.

##### Boolean paths

The asymmetric BIRs provide a unique dimension to the network that is fundamentally different from any other gene expression networks in the literature. Traversing a set of nodes in a directed graph of the Boolean network constitutes a Boolean path that can be interpreted as follows. A simple Boolean path involves two nodes and the directed edge between them. This simple Boolean path can be interpreted as shown in the supplementary figure (Supplementary Fig. S1E). For the nodes X and Y with X low => Y low only quadrant #1 is sparse; the other quadrants #0, #2, and #3 are filled with samples (Supplementary Fig. S1E). Assuming monotonicity in X and Y, the quadrants can be ordered in two possible ways: 0-2-3 and 3-2-0. The path corresponds to 0-2-3 begins with X low and Y low. This is interpreted as X turns on first and then Y turns on along a hypothetical biological path defined by the sample order. Similarly, Y turns off first and then X turns off in the path 3-2-0. A complex path in the Boolean network involves more than one Boolean implication relationship (Supplementary Fig. S1F). Three Boolean implication relationships can be used to group samples into five bins and the bins can be ordered in two possible ways (Supplementary Fig. S1F, forward, reverse). Another example of a path is illustrated in supplementary figure (Supplementary Fig. S1G).

##### Discovery of paths in clustered Boolean implication network

We focus on paths that are transitive (such as Supplementary Fig. S1F and G) because they represent a simple change in gene regulation, i.e., going from low-to-high or high-to-low once along a path (See *Boolean paths* above). By contrast, complex change refers to changes of gene regulation multiple times along a path such as a gene going from high-to-low and then back to high. Discovery of paths start with a node that represents the biggest cluster in the CBIN. Since a path of high => high, high => low, and low => low can be used to order samples as shown in Supplementary Fig. S1F, we try to identify paths of this type that intersects the big clusters (top 5, based on size) in the network. To maintain the transitivity this path can be expanded as the chain of high => high, followed by high => low, followed by another chain of low => low. We would like to keep one high => low in a path because that will cover genes that are both up- and down-regulated. Since, the path A high => B high can also be written as B low => A low, the chain of high => high can be reduced to the chain of low => low in reverse direction. Therefore, we must focus only on the high => low and chain of low => low. We developed a simple, intuitive algorithm that traverses the nodes of the CBIN starting with the biggest cluster and greedily chooses next big cluster connected to the nodes visited in sequence. The emphasis on cluster sizes comes from the fundamental assumption that size determines importance and relevance. Therefore, we start from a big cluster (A1 from the top 5) and identify other clusters that form a chain of low => low. Further, we identify other clusters that are either opposite to A1 or they have high => low relationship with A1, and the biggest cluster (A2) among these clusters were chosen. In addition, a chain of low => low relationship from A2 is identified. In each subsequent step, again the biggest cluster among the different choices was greedily chosen. Finally equivalence relationship from each cluster is used to gather more genes in each cluster and the whole path is clustered based on equivalence relationships. Depth-first traversal (DFS) was used to follow the path of low => low where bigger clusters are visited first. The search was performed until a cluster was reached for which there is no low => low relationships. For example, starting with cluster S, the search will return S low => A1 low, A1 low => A2 low, and A2 low => A3 low if A3 doesn’t have any low => low relationships. Similarly, a new starting point is considered S2 such that S2 is the biggest cluster X that has either S high => X low or S Opposite X. From cluster S2 another DFS was performed to retrieve the longest possible path of low => low. The search may return S2 low => B1 low, B1 low => B2 low if B2 doesn’t have any low => low relationships. In summary, the most prominent Boolean path was discovered by starting with the largest cluster and then exploring edges that connected to the next largest cluster in a greedy manner. This process was repeated to explore paths that connect the big clusters in the network.

##### Scoring Boolean path for sample order

A composite score was computed for a specified Boolean path that can be used to order the sample which was consistent with the logical order. To compute the score, first the genes present in each cluster were normalized and averaged. Gene expression values were normalized according to a modified Z-score approach centered around StepMiner threshold (formula = (expr − SThr)/3∗stddev; Supplementary Fig. S2B). Weighted linear combination of the averages from the clusters of a Boolean path was used to create a score for each sample. The weights along the path either monotonically increased or decreased to make the sample order consistent with the logical order based on BIR. The samples were ordered based on the final weighted (−1 for C#13, 1 for C#14 and 2 for C#3) and linearly combined score (Supplementary Fig. S2C). The direction of the path was derived from the connection from a reactive cluster to a tolerant cluster. The sample order is visualized by a color-coded bar plot and a violin + swarm plot (Supplementary Fig. S2C). A noise margin is computed for this composite score which follows the same linearly weighted combined score on 2-fold change ( ± 0.5 around StepMiner threshold).

##### Summary of genes in the clusters

Reactome pathway analysis of each cluster along the top continuum paths was performed to identify the enriched pathways.^32^ The pathway description was used to summarize at a high-level what kind of biological processes are enriched in a particular cluster. List of genes and the pathways enriched in them are provided in Supplemental Information 2. Clusters 13, 14, 3 list of genes are ranked based on equivalences within the cluster and the differential expression between M1 and M2.

##### Cross-species gene name conversion

Orthologous human and mouse genes were identified using ensemble GRCh38.p13-100 gene annotations. Human to mouse gene name conversion and vice-versa used this database.

##### Machine learning to discover models of macrophage polarization

We implement supervised learning in which we use labelled training data of extremes of macrophage polarized states to train a model that can recognize a continuum of diverse functional states during macrophage polarization. Briefly, to identify gene regulatory changes during macrophage polarizations from M0 to M1 and/or M2, we employed the MiDReG (Mining Developmentally Regulated Genes) algorithm, which utilizes statistical learning techniques.^30^^,^^33^ By applying statistical model checking to Boolean invariant rules within a static cross-sectional dataset, MiDReG infers the underlying temporal events. It identifies temporal logical changes in gene regulation by exploring transitive Boolean paths (Supplementary Fig. S1E–G). We applied the MiDReG algorithm to analyse large and diverse macrophage datasets (GSE134312), discovering Boolean invariant rules and constructing a clustered Boolean Implication Network. The model was trained by labelling the macrophage polarization states in GSE134312 as M0 (n = 47), M1 (n = 13) and M2 (n = 8) based on ligand treatments that are well-established as stimuli for driving either M1 (LPS, IFNγ) or M2 (IL4, IL13) states (See Supplementary Table S1). The algorithm takes the macrophage network (selected graph) and this labelled dataset GSE134312 as inputs and identifies the best model to recognize the labels (See function learningAlgorithm in github codebase BoNE/SMaRT/MacUtils.py and the outputs in BoNE/SMaRT/macrophage.ipynb). The algorithm enables a comprehensive search for macrophage polarization states based on transitive Boolean paths that contains three nodes with one high => low relationships. The high => low relationships cover both up/down regulated genes and additional Boolean path of high => high or low => low provides features to improve predictions. An unbiased search for these patterns results in 7 different Boolean paths [1, 2, 3], [1, 14, 3], [12, 5, 6], [11, 10-8], [13-14-3], [10, 8, 9], and [1, 12, 5]. The nodes on the high => high side were assigned negative weights (−2, −1, etc.) and the nodes on the low => low side assigned positive weights (1, 2 etc.) to compute an optimal composite score. Three different ROC-AUCs were computed (M0, M1 and M2) to measure the performance of the composite scores for the Boolean paths. ROC-AUC of M1 and M2 were multiplied together that represent overall performance of a Boolean path. Performance of Boolean path 13-14-3 was better than all other paths.

##### Signatures of macrophage reactivity and tolerance (S-Ma-R-T) computation

*BoNE* uses Boolean implication network on macrophage dataset to build a signature of macrophage polarization. Selected clusters by size connected by high => high (green arrow), high => low (red arrows) and low => low (blue arrows) Boolean implication relationships. Reactome analysis of each clusters shows the biological processes the genes are involved in (Supplementary Fig. S2A). A path is selected in the network that is used to test M1/M2 states classification. This process is demonstrated by using a path #13-14-3 on GSE134312 (Supplementary Fig. S2B and C).

##### Single cell data analysis

Single cell datasets were processed using scanpy (v1.5.1) framework. Composite scores for C#13 (weight = −1) and C#14-3 (weights = 1, 2) were computed like bulk RNASeq datasets. Scatterplot between C13 and C14-3 score were plotted using pandas plotting functions. StepMiner threshold is computed for C#13 and C#14-3 composite scores and display as vertical and horizontal lines in the scatterplots. Bottom-left quadrants enrich reactive and top-right quadrants enrich tolerant macrophages based on our *BoNE* derived models.

##### Normalization of gene expression based on circadian rhythm

Since the state of macrophage swings from reactive to tolerant from day to night^34^ (See Supplementary Fig. S3), it is important to control for this variation during analysis of macrophage polarization. To start the normalization process, clock genes (such as DBP, ARNTL, etc.) or gene signatures that capture circadian rhythm is used to adjust the *BoNE* score (Supplementary Fig. S4). First, both the *BoNE* score (Supplementary Fig. S4B) and the clock gene expression are scaled for each sample type based on their dynamic range of expression values (min – max). For example, the dataset GSE98895 contains two sample types: C (Control), and MetS (Metabolic Syndrome). Let’s take one sample from the MetS group (x, y) where x is the clock gene expression value and y is the original *BoNE* score (Supplementary Fig. S4C). Bounding box for the MetS group demonstrates the range of values for both the *BoNE* score (S1) and the clock gene expression (S2). An average of *BoNE* scores and the clock gene expression is shown using an orange diamond. The distance of (x, y) from the orange diamond (S3, S4) is used to scale both values (x − S3∗(S2 + 1)/(S1 + 1), y + S4∗(S1 + 1)/(S2 + 1)). This process is repeated using control (C) samples using the green diamond. Linear regression is used to compute the trend between the transformed *BoNE* score and clock gene expression (y = mx + c; Supplementary Fig. S4D). The trend is subtracted from the transformed *BoNE* score to compute the final normalized *BoNE* score (y = mx − c). Samples are now rank ordered based on the final normalized *BoNE* score to visualize the effect of normalization process.

##### Proteomics analysis

A multiplexed TMT (tandem mass tags) quantitative proteomics dataset has been obtained from He, L. et al.^35^ (see Key Resource Table). To generate this dataset, authors had differentiated human THP-1 cells with phorbol myristate acetate (PMA) for 24 h into macrophages (M0 state). The M0 cells were subsequently treated with IL4 for M2 polarization and with LPS and IFNγ for M1 polarization over a 24-h time-period. Samples were processed for quantitative mass spectrometry at 1 h, 4 h, 8 h, and 24 h. Ratio of raw intensity values has been compared between M1 and M2 states to obtain the list of induced proteins at various time points (see Supplemental Information 3). To obtain the list of proteins induced in M1 state, the cut-off used for induction of proteins when comparing the raw intensity ratio for LPS/IFNγ over IL4 stimulation for all time points was ≥2. To obtain the list of proteins induced in M2 state, the cut-off used for induction of proteins when comparing the raw intensity ratio for IL4 over LPS/IFNγ stimulation for all time points was ≥ 1.5.

To assess the differential enrichment of proteins across different signatures for both M1 and M2 polarization states at various time points, we used the following equation to calculate the z-test of proportions,

$$z=\frac{\left(p1-p2\right)}{\sqrt{p\left(1-p\right)\left(\frac{1}{n1}+\frac{1}{n2}\right)}}$$

Here, p1 is sample proportion (x1/n1) of proteins translated from the “reactive” signature that were induced ≥2 fold upon LPS stimulation. And p2 is the sample proportion (x2/n2) of proteins translated from the “tolerance” signature that were induced ≥1.5 fold upon IL4 stimulation. Here, p = (x1 + x2)/(n1 + n2).

##### Ethics statement

No ethical approval was required as our study design incorporated publicly available datasets.

##### Role of funders

The funders had no role in the study design, data collection and analysis, decision to publish, or preparation of the manuscript.

## Results

### A computational model of continuum states in macrophage processes

We chose a Boolean approach to build transcriptomic network^29^; this approach has been used to create maps of evolving cellular states along any disease continuum and identify cellular states in diverse tissues and contexts with high degrees of precision (see detailed Methods). The Boolean approach relies on invariant relationships that are conserved despite heterogeneity in the samples used for the analysis. Invariant relationships among pairs of genes that are conserved across samples representative of maximum possible diversity, i.e., irrespective of their origin (normal or disease), laboratories and/or cohorts, different perturbations, are assumed to be fundamentally important for any given process.

For model training and development, we used a pooled all-human microarray dataset that included 197 manually annotated heterogeneous macrophage datasets from GEO (GSE134312^36^; Fig. 1a–c; Supplementary Fig. S1A and B; see Supplemental Information 1 for catalogue of datasets). These datasets contained primary tissue-derived macrophages (both healthy and diseased tissues) and cultured macrophage cell lines (e.g., THP1), either untreated or treated with diverse sets of ligands that are known to induce either M1 (n = 13) or M2 (n = 8) polarized states (see Supplementary Table S1).

A graph (Fig. 1d and Supplementary Figs. S1C and S2A) is built, comprised of gene clusters (nodes) connected to each other using Boolean implication relationships (edges). The network displayed scale-free properties, as expected (Supplementary Fig. S1D). We oriented ourselves to the resultant network by querying and locating the known ‘M1/M2’ samples; the ‘M1’ samples segregated towards one end, and ‘M2’ samples on the other, implying that the paths of connected clusters within the resultant network represent a continuum of cellular states in macrophages within the immunologic spectrum (Supplementary Fig. S1E–G). Reactome pathway analyses^32^ of each cluster along the top continuum paths revealed a multitude of cellular processes that are impacted during macrophage polarization (Fig. 1e and Supplemental Information 4; Gene clusters and reactome pathways can be queried at: http://hegemon.ucsd.edu/SMaRT/).

### Identification of signatures of macrophage ‘reactivity’ and ‘tolerance’ (SMaRT)

Next, various interconnected gene clusters (i.e., Boolean paths) were assessed for their ability to accurately classify the samples (based on the genes in the clusters and computing a weighted average of gene expression values outlined in Supplementary Fig. S2B) (Fig. 1f). A multivariate analysis of the top five Boolean paths revealed that the path connecting clusters(C)#13 → 14 → 3 is the best (p < 0.001) at discriminating M1 (ROC-AUC 0.98) and M2 (ROC-AUC 0.99) (Fig. 1f and Supplementary Fig. S2C). Path #13 → 14 → 3 was subsequently validated in five other independent datasets (Fig. 1g). A comparative analysis of #13 → 14 → 3 path vs other traditional approaches, e.g., Differential Expression,^37^ Correlation Network,^37^ Hierarchical Clustering^38^ and Differential and interactome analyses^39^ showed the superiority of the *BoNE*-derived path in separating M0-M1-M2 states. The Boolean path matched differential expression in its ability to distinguish M1 state, while exceeding the remaining traditional approaches (Fig. 1h and Supplementary Fig. S5). A heatmap of the pattern of gene expression in each cluster in M0-M1-M2 states is shown in Fig. 1i.

Furthermore, C#13 predicted M1 perfectly (ROC-AUC = 1.00) and the path #14 → 3 predicted M2 close to perfection (ROC-AUC = ranging from 0.80 to 1.00) in all cohorts tested (Fig. 1j). This indicates that while the path #13 → 14 → 3 is the most accurate path across all human macrophage-derived datasets collected and analysed, C#13 and the path #14 → 3 carry relevant information on macrophage states independently of each other. C#13 is associated with M1-like state and expression of these genes is predicted to reflect the extent of “immunoreactivity” of macrophages. Path #14 → 3 is associated with a M2-like state and expression of these genes is predicted to reflect the extent of “immunotolerance”. We define the two distinct macrophage polarization states in physiology as “reactive” and “tolerant” based on basal C#13 and #14 → 3 scores, respectively (Fig. 1k). Four additional macrophage states could also exist, presumably in disease states, i.e., hyperreactive (high C#13), hyper tolerant (high #14 → 3), hyporeactive (low C#13), and hypo tolerant (low #14 → 3) (Fig. 1k). Henceforth, we refer to these genes as *s*ignatures of *ma*crophage *r*eactivity and *t*olerance, abbreviated as ‘*SMaRT’* (See http://hegemon.ucsd.edu/SMaRT/and Supplemental Information 2 for the list of genes, ranked based on their log2 fold change between M1 vs M2 human macrophage samples in our training dataset, GSE134312).

### SMaRT genes are relevant across tissues, organs, species, and immune cells

We found that the path #13 → 14 → 3 successfully identified M1/M2-polarization states in diverse tissue-resident macrophages (brain-resident microglia, the Langerhan’s cells in the skin, intestinal and lung alveolar macrophages, etc.), in both humans and mice (Fig. 2a and b). See Supplemental Information 1 for the degree of heterogeneity represented in these datasets. Surprisingly, the path could also separate reactive and tolerant states of other immune cells, including lymphocytes (B/T and NK-T), natural killer (NK) cells, neutrophils, dendritic, basophils, eosinophils, and mast cells (Fig. 2c and Supplementary Fig. S2H). Together, these findings indicate that the *SMaRT*-based definitions of ‘reactivity’ and ‘tolerance’ remain relevant in the context of tissue-resident macrophages despite their adaptation to the tissue and/or organ-specific microenvironment for their identity.^40^, ^41^, ^42^ These definitions also maintain relevance in mice, whose immune system is different from ours.^43^ Findings suggest that the *SMaRT*-based definitions may reflect the *fundamental* immune-reactive and tolerant gene regulatory mechanisms that are shared among diverse cells in our immune system, regardless of whether they are derived from the myeloid or lymphoid lineage (Fig. 2d).

### The network captures physiologic macrophage states and functions

We found that our model of macrophage processes includes several well-defined macrophage subtypes (Supplementary Fig. S2I). The classical M1 subtype was represented in C#1 and #13 on the reactive end of the model, alongside TCR+ macrophages in C#1 and #12; the latter is known to release CCL2 and have high phagocytic abilities.^44^ On the tolerant end of the model, we found the TAMs in C#2, #5, #6, and the *CD169*+ macrophages in C#2, #3, and #7; both subtypes have been implicated in immunological tolerance.^45^, ^46^, ^47^ As one would anticipate, the tissue-resident macrophages (M2a-d) that are known for their plasticity of polarization states were more centrally placed in C#2 and #5. Finally, gene signatures of scar-associated non-inflammatory (ni) macrophages that restrict inflammation in liver cirrhosis (SAM B^12^ and SAM ni,^13^ Fig. 2e) and damage-associated microglia (DAMs^10^; Fig. 2e) that restrict the progression of neurodegeneration significantly overlapped with the tolerant clusters C#14 and #3. A gene signature that was recently shown to be induced in monocytes and macrophages in all viral pandemics^48^ (ViP), past and present, overlapped as expected, with the reactive C#13 (Supplemental Information 2 lists all gene signatures in Fig. 2e).

Members of the family of pattern recognition receptors (PRRs; Supplementary Table S2), via which macrophages ‘sense’ its surroundings,^49^ were distributed in various nodes within the model, overlapping with each other (Supplementary Fig. S2J). PRRs that sense pathogens or apoptotic cells to stimulate phagocytosis and mediate inflammation, e.g., toll-like (TLRs), nucleotide oligomerization domain (NODs) and receptor for advanced glycation end products (RAGE) were found on the ‘reactive’ side of the model. The TLRs, scavengers and C-type lectins also overlapped with path#13 → 14 → 3, but only on the tolerant end (cluster #3) of the spectrum.

The circadian genes were distributed within clusters along a path (#1 → 2→3 → 4) (Supplementary Fig. S2K), intersecting at the tolerant end of the path#13 → 14 → 3, i.e., C#3. The daytime circadian genes were in the reactive end of the model and showed an inverse high => low Boolean relationship with night-time circadian genes; the latter were mostly in the tolerant end of the model (Supplementary Fig. S3A–C). This finding is consistent with the current belief that macrophages ‘kill’ (react) during the day and ‘heal’ (tolerate) during the night.^50^ We also show that the performance of the tolerant signature (C#14-3) in diseases that have an intricate relationship with circadian rhythms, such as metabolic syndrome,^51^ can be further improved by normalization based on a clock gene or clock gene signature (Supplementary Fig. S4).

It is noteworthy that while C#13 is associated with ‘reactivity’, C#14 and C#3 are associated with ‘tolerance’, the other clusters do not clearly represent either state. Reactome pathway analysis for the remaining clusters showed: C#1 (3213 genes) is the biggest cluster with no significant pathways; C#2 (2448 genes) is enriched in SUMOylation-related processes; Clusters C#4, C#6, C#8, C#9, C#10, and C#12 have no significantly enriched pathways; C#5 (Viral Infection, Nonsense-mediated decay), C#7 (WNT signalling), C#11 (Gap Junction), C#13 (Immune System), C#14 (Viral Infection, Nonsense-mediated decay) (See Supplemental Information 4).

### SMaRT genes identify polarization states at single cell resolution

An analysis of composite expression scores of genes in C#13 vs path #14 → 3 revealed a consistent pattern of macrophages polarized towards M1 and M2 in multiple independent single cell RNA Seq (scSeq) datasets (Fig. 2f and g and Supplementary Fig. S2D–G) in both human (GSE168710, GSE164498, Fig. 2f) and mouse (GSE161125, GSE158094, Fig. 2g). A StepMiner threshold (blue lines, Fig. 2f and g) is computed for both C#13 and path #14 → 3 composite scores that divide the scatterplots into four different quadrants. Bottom-left quadrant shows a significant enrichment of reactive macrophages (M1) in all four scatterplots (p < 0.001, Fig. 2f and g). Traditional UMAP analyses in the above datasets show distinct clusters for the different polarized states (Fig. 2h) but it is hard to translate that in Crohn’s disease (CD) dataset (GSE134809, Fig. 2i*-top*). Since *BoNE* derived signatures show significant enrichment of the reactive macrophages in the bottom-left quadrant (as shown in Fig. 2f and g), it can easily be tested in CD dataset. As expected, in the bottom-left quadrant (reactive macrophages) of the CD dataset (Fig. 2i*-bottom*), macrophages from involved tissues are significantly enriched (p < 0.001) compared to uninvolved whereas no significant enrichment (p = 0.08) was observed between ileal vs blood tissue. These findings are consistent with macrophage phenotypes observed in inflammatory bowel disease patients.^52^^,^^53^ The SMaRT genes were also able to recognize differential enrichment of reactive and tolerant macrophages in scSeq studies of yet another complex, chronic inflammatory condition of multicellular origin, i.e., pulmonary fibrosis (GSE122960) (Supplementary Fig. S6).

Furthermore, to test if presence of both reactive and tolerant states can be detected by *BoNE* models, we artificially created pseudobulk samples using various proportions of M1 and M2 cells, and one sample with 30% M1 and 30% M2 cells (Mixed) in the background of lung cells from a scSeq dataset GSE150708 (human, Supplementary Fig. S2D). The ‘Mixed’ sample was categorized as both tolerant and reactive as expected using C#13 and path #14-3 signatures (Supplementary Fig. S2D). Blood from CD-afflicted subjects (GSE34809, Supplementary Fig. S2G) was categorized as both tolerant and reactive, which could reflect their proinflammatory state in the setting of impaired microbial clearance.^54^

### SMaRT genes identify pathologic polarization states in diseases

**Fig. 3:**
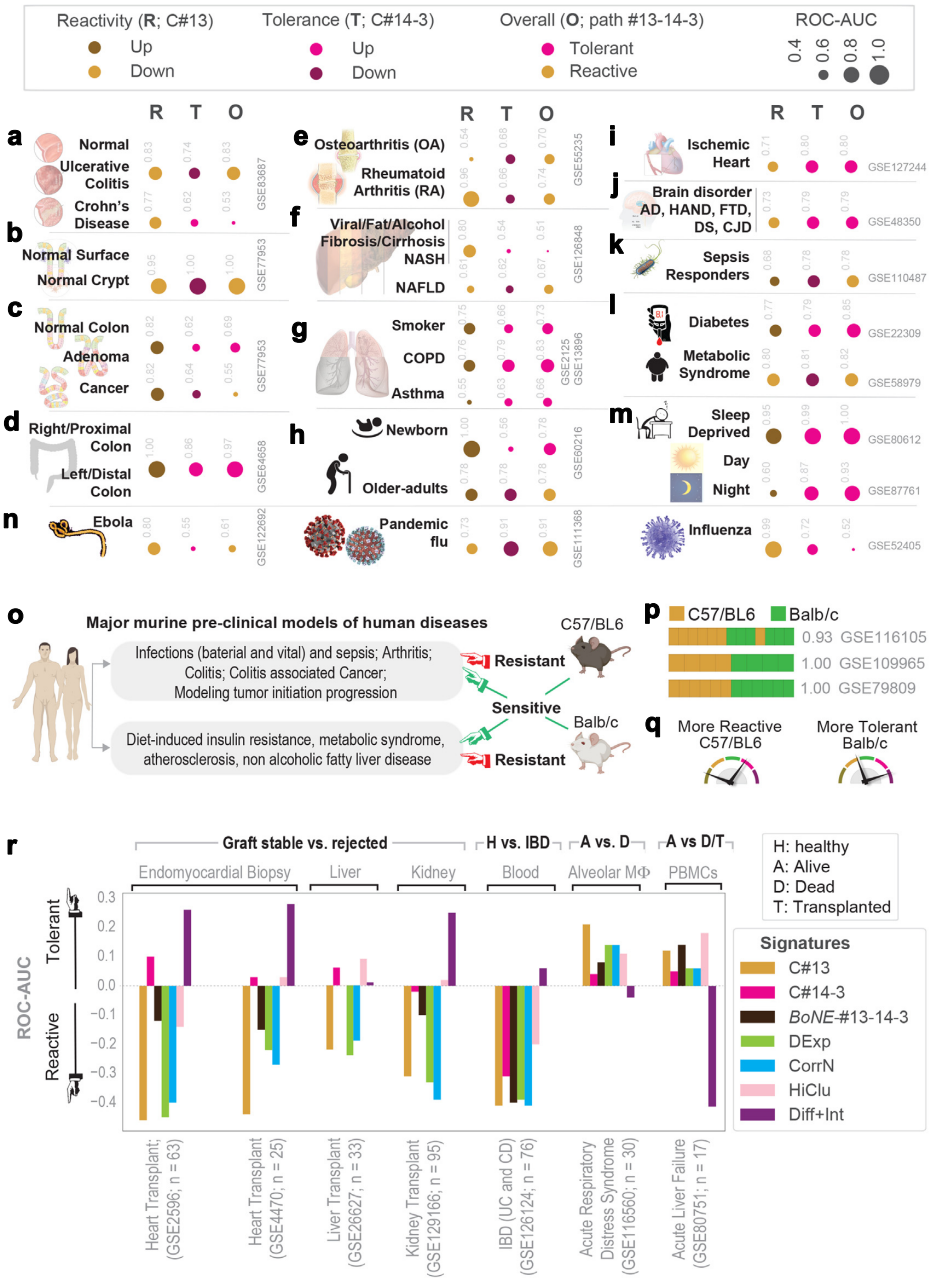
**Definitions of “reactivity” and “tolerance” detects pathologic macrophage states in disease.** Tissue immune microenvironment is visualized (in panels a–n) as bubble plots of ROC-AUC values (radii of circles are based on the ROC-AUC; Key on top) demonstrating the direction of gene regulation (Up vs Down; Key on top) for the classification of samples using BoNE-derived gene signatures of either reactive (R; C#13) or tolerant (T; C#14-3) or overall (O; path #13 → 14 → 3) in columns. The ROC-AUC values are provided next to the bubble. Sample diversity and sizes are as follows: **a)** IBD; GSE83687, n = 134; 60 Normal, 32 Ulcerative Colitis, 42 Crohn’s Disease. **b)** Colon crypt; GSE77953, 6 Normal Surface vs 7 Normal Crypt base. **c)**; Colon cancer: Pooled colon dataset from NCBI GEO; n = 170 Normal, 68 Adenomas, 1662 CRCs. **d)** Colon anatomy: Proximal (right) vs distal (left) normal colon from mouse (GSE64423, n = 6) and human (GSE20881, n = 75). See Supplementary Fig. S7 for violin plots. **e)** Arthritis; GSE55235, GSE55457 and GSE55584, n = 79; 20 Normal, 33 Rheumatoid Arthritis, 26 Osteoarthritis. **f)** Hepatitis: GSE89632, n = 63; 20 fatty liver, 19 Non-alcoholic steatohepatitis (NASH) and 24 healthy, alcoholic liver disease (GSE94417, GSE94397 and GSE94399, n = 195; 109 Healthy, 13 Alcoholic Hepatitis, 6 Alcoholic fatty liver (AFL), 67 Alcoholic cirrhosis (AC) and viral hepatitis (GSE70779, n = 18; 9 Pre-treatment, 9 Post-treatment with direct-acting anti-virals). **g)** Chronic lung disease; GSE2125 and GSE13896, n = 115; 39 Non-smoker, 49 Smoker, 15 Asthma, 12, Chronic Obstructive Pulmonary Disease (COPD). **h)** Aging process; GSE60216, n = 9; 3 Newborn babies, 3 Adults, 3 Old-adults. **i)** Cardiomyopathy (CM), ischemic and non-ischemic (I/NI); GSE104423, n = 25 human samples; 14 NICM, 11 ICM; GSE127244, n = 24 mouse samples, 16 NICM, 8 ICM. **j)** Neurodegenerative brain disorders; GSE118553 (n = 401) and GSE48350 (n = 253), Alzheimer’s disease (AD); GSE35864, HIV-associated neurocognitive disorder (HAND; n = 72); GSE13162, frontotemporal dementia (FTD; n = 56); GSE59630, Down’s Syndrome (DS; n = 116); GSE124571, Creutzfeldt-Jakob Disease (CJD; n = 21). **k)** Systemic inflammatory response syndrome (SIRS) and sepsis; GSE63042 (n = 129); GSE110487 (n = 31). **l)** Type 2 diabetes and metabolic syndrome; GSE22309 (n = 110), Pre- and post-insulin treatment muscle biopsies from 20 insulin sensitive, 20 insulin resistant, 15 T2DM; GSE98895 (n = 40), PBMCs from 20 control, 20 metabolic syndrome. **m)** Sleep deprivation and circadian rhythm; GSE9444, n = 131 mouse brain and liver samples; GSE80612, twin, n = 22 human peripheral blood leukocytes; GSE98582, n = 555 human blood samples; GSE104674, n = 48, 24 healthy and 24 T2DM. **n)** Viral pandemics, such as SARS, MERS, Ebola, and others [see Supplementary Fig. S9E]. See Supplementary Fig. S8 for violin plots relevant to panels **e–j**. See Supplementary Fig. S9 for violin plots relevant to **k–m**. **o–q)** Schematic (**o**) summarizes the use of two major mouse strains (C57/B6 and Balb/c) commonly used for modeling two broad categories of human diseases. Bar plots (**p**) showing sample classification of genetically diverse macrophage datasets based on expression levels of genes in C#13. Schematic (**q**) summarizes findings. **r)** The diagnostic potential of various indicated gene signatures were tested on multiple datasets generated from tissues derived from patients with the known clinically relevant outcome, as indicated. In each case, BoNE-derived signatures were compared against four traditional approaches.

To determine how the Boolean network-derived *formal* definitions perform in disease states, we analysed a plethora of disease conditions and tissues (Fig. 3a–n and Supplemental Information 1). We computed a composite immune response score derived from C#13 alone or C#14 and #3, which quantitatively estimates the degree of “reactivity” and “tolerance”, respectively, and tested it in diverse conditions. An analysis of full-thickness colon tissues representing the 2 major subtypes of inflammatory bowel disease (IBD), ulcerative colitis (UC) and Crohn’s disease (CD) (Supplementary Fig. S7A) revealed that reactivity is a common feature in both UC and CD (Fig. 3a, *top*; Supplementary Fig. S7B-*left*). However, tolerance was enhanced only in CD (Supplementary Fig. S7B*-right*), which is consistent with the notion that ‘alternatively’ activated tolerant macrophages may drive the transmural nature of the inflammation, ineffective bacterial clearance, and accompanying tissue remodelling (fibrosis, stricture, fistula), all features that are observed uniquely in CD,^55^ but not UC. Reactivity alone could prognosticate outcome (i.e., segregate responder vs non-responder) regardless of the heterogeneity of the UC cohorts and the diverse treatment modalities (Supplementary Fig. S7C and D), consistent with the widely-accepted notion that hyperinflammatory macrophages are drivers^56^ of the disease and key targets for therapeutics.^57^ Insufficient datasets precluded similar analyses in the case of CD.

We also found that “reactivity” and “tolerance” differs along the length of the colon crypt—the surface is more reactive, whereas the stem-cell niche at the bottom is more “tolerant” (Fig. 3b and Supplementary Fig. S7E and F). We also found that “hypo-reactivity” [low C#13] and “complete tolerance” [high #14 → 3] are two states that are progressively accentuated during colorectal carcinoma (CRC) initiation and the emergence of chemoresistance (Fig. 3c and Supplementary Fig. S7G and H). Consistent with the fact that most of the CRCs are found located in the left (distal) colon and microbe-driven risk is high in that segment,^58^ we found that segment to be more tolerant than the right (proximal) segment (Fig. 3d).

We detected altered macrophage states during the initiation and progression of several human other diseases, ranging from arthritis, through neurodegenerative diseases to viral pandemics (see Fig. 3e–n and Supplementary Figs. S8A–N and S9A–E). Our definitions for “reactivity” and “tolerance” could accurately identify the underlying pathologic macrophage states implicated in each condition. Together, these results show that the *BoNE*-derived signature can detect different subsets of macrophages are essential to the pathogenesis of many diseases. Findings also agree with the notion that disease chronicity is invariably associated with mixed polarization states (whose detection has largely been enabled by scSeq studies) where each state plays an opposing (balanced) role.^2^^,^^8^, ^9^, ^10^, ^11^, ^12^, ^13^

### SMaRT genes rationalize the choice of mouse models

Although mice are the preferred model species for research,^59^ most agree that their innate immune systems differ.^43^ C57BL/6J and Balb/c mice are two most used mouse strains that differ in their immune responses, giving rise to distinct disease outcomes, which in turn rationalizes their use as pre-clinical models for human diseases (Fig. 3o). Our signature successfully classified the macrophages from these two strains in three independent cohorts^60^^,^^61^ (Fig. 3p); C57BL/6 emerged as more reactive and Balb/c as more tolerant (Fig. 3q). These findings are consistent with the observation that BALB/c mice are more susceptible to a variety of pathogens,^62^, ^63^, ^64^ and are useful for modelling tumour initiation and progression and for making antibodies. By contrast, C57BL/6 mice are resistant to infections and are the most common strain used for modelling inflammatory diseases, e.g., arthritis, metabolic disorders [NASH, atherosclerosis, etc.^65^, ^66^, ^67^]. We conclude that the model-derived definitions for “reactivity” and “tolerance” —(i) capture the contrasting immunophenotypes of these two murine strains previously reported by Mills et al.,^3^ and (ii) rationalize the choice of each strain as preferred models for modelling a unique set of human diseases. Findings also suggest that the model-derived signatures could serve as an objective guide for assessing the appropriateness of any species/strains/sub-strains as pre-clinical models.

### SMaRT genes carry diagnostic value

Next we compared head-to-head the diagnostic and prognostic potential of the newly defined polarization states against four traditional definitions: differential expression analysis^37^ (DExp), correlation network^68^ (CorrN), hierarchical clustering + fold change^38^ (HiClu), and differential + interactome analysis^39^ (Diff + Int). A composite immune response score derived from C#13 alone, which quantitatively estimates the degree of “reactivity” was tested on multiple datasets generated from tissues derived from patients with known clinically relevant diagnoses. A hyper-reactive state was invariably associated with graft rejection in transplanted hearts, livers, and kidneys (Fig. 3r). A ‘hyper-reactive’ state also classified IBD-afflicted children from those with non-IBD indications (8–18 y age) with reasonable accuracy in a prospective study where the blood samples were drawn at the time of diagnostic colonoscopy (Fig. 3r). Among the critically ill patients in the ICU, a hyper-reactive state was associated with better 28-day survival for those with ARDS on ventilators (Fig. 3r) and improved survival without the need for liver transplantation in those diagnosed with Tylenol-induced acute liver failure (Fig. 3r). While some of the four other traditional methodologies fared similar to the new definitions in some cohorts, none performed as well, and/or as consistently. Findings suggest that the *BoNE*-derived signatures may capture fundamental aspects of macrophage polarization that drive disease states.

### SMaRT genes can prognosticate outcome

**Fig. 4:**
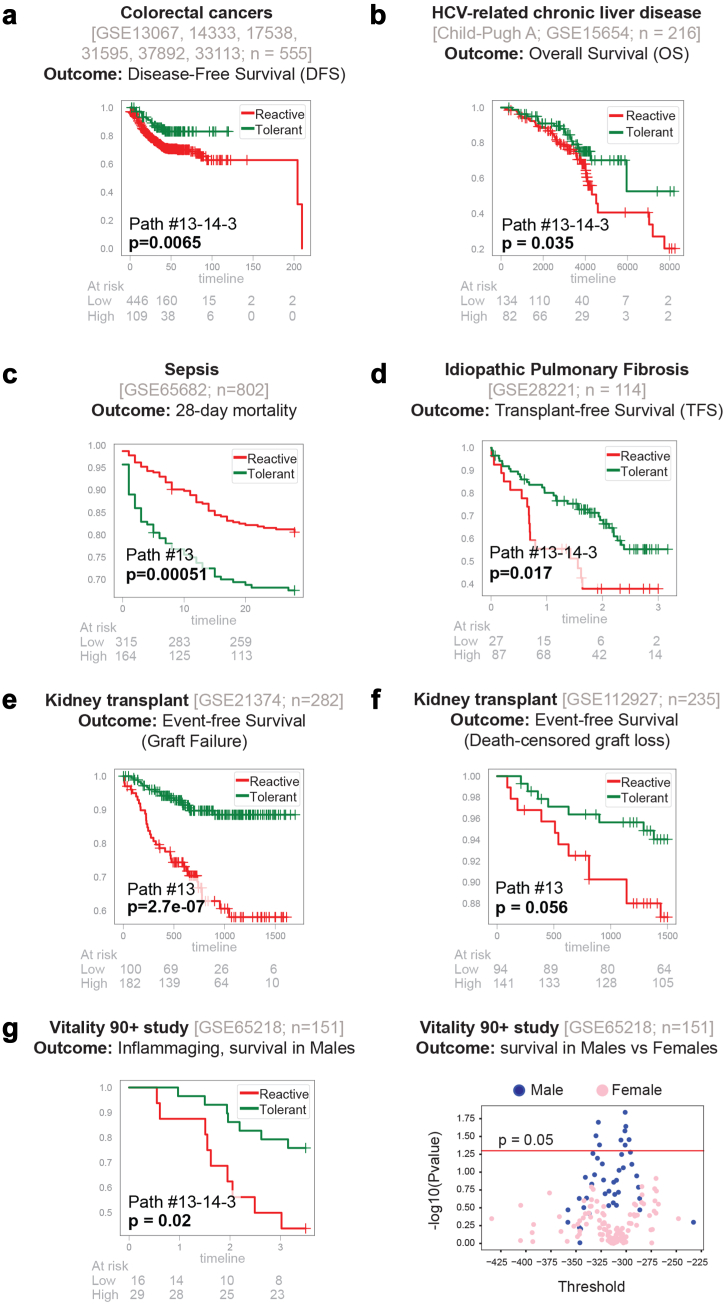
**Prognostic potentials of *SMaRT genes*. a–g)** The prognostic performance of the *BoNE*-derived *SMaRT* genes is evaluated across diverse disease conditions (colon cancer, **a**; liver fibrosis, **b**; sepsis, **c**; idiopathic pulmonary fibrosis, **d**; kidney transplantation, **e and f**; inflammaging, **g-*left***). Results are displayed as Kaplan Meier (KM) curves with significance (*p* values) as assessed by log-rank-test. A composite immune response score is computed using Boolean path #13 → 14 → 3 or C#13 alone, as indicated within each KM plot. Low score = “reactive”; high score = “tolerant”. A threshold is computed using StepMiner by searching three options (thr, thr ± noise margin) on the immune score to separate these two states. **g-*right***) Scatterplot between all possible thresholds of the #13 → 14 → 3 composite score and -log10 of the p value from the log-rank test for both male (blue) vs female (pink) separately. Pvalues are significant above the red line (*p* = 0.05). See also Supplementary Fig. S9F for other cancers (breast, prostate, pancreas, glioblastoma, and bladder).

We next computed a composite immune response score based on either the path #13-14-3 or C#13 alone. When used as a composite score, a low score value represents “reactive” and high score value represent “tolerant” states. This signature was tested on all transcriptomic datasets found on the NCBI GEO database (as of *04/2022*) originating from prospective studies, regardless of disease. Prospective studies were chosen because they rarely have selection bias from enrolment procedures because the outcomes have not yet occurred at the time of enrolment. In the context of cancers, “reactive” tumours carried a worse prognosis than “tolerant” ones across a variety of solid tumour subtypes, e.g., colorectal (n = 555; Fig. 4a), breast, pancreas, prostate, glioblastoma and bladder cancers (Supplementary Fig. S9F). These findings are consistent with the well-recognized role of inflammatory cells in the tumour microenvironment.^69^

In a cohort of 216 patients with HCV-related liver fibrosis, overall survival was reduced among patients with a “reactive” signature on their liver biopsies compared to those with a “tolerant” signature (Fig. 4b). These findings are consistent with the known role of activated macrophages in chronic liver injury, inflammation and fibrosis.^70^, ^71^, ^72^, ^73^

In a cohort of 802 patients with sepsis, 28-day mortality was worse among those with a “tolerant” signature compared to those with a “reactive” signature (Fig. 4c). This finding is consistent with the notion that “endotoxin tolerance” during sepsis carries poor outcome.^74^

In a cohort of 114 patients with idiopathic pulmonary fibrosis (IPF), an incurable disease that is characterized by progressive fibrosis requiring lung transplantation,^75^ a “reactive” signature was associated with shorter transplant-free survival (Fig. 4d). Results are in keeping with the widely accepted notion that proinflammatory pulmonary macrophages are known to drive inflammation and fibrosis in the lung.^76^

Among 517 recipients of kidney transplants, a “reactive” signature was associated with increased graft loss in two independent cohorts (Fig. 4e–f). Findings are in keeping with prior body of work implicating inflammatory macrophages (both number and extent of activation) as culprits in both acute and chronic allograft rejection and graft loss.^77^, ^78^, ^79^

Finally, among 151 nonagenarians in the Vitality 90+ study,^80^ a “reactive” signature was associated with higher mortality in men (Fig. 4g-*left*). No significant results were found in women (Fig. 4g-*right*). Results are in keeping with the fact that the plasma levels of the ‘classical’ marker of inflammaging, i.e., interleukin-6 (IL-6) and a pro-inflammatory gene signature in PBMCs were correlated in men, whereas no correlations were observed in women.^81^

These findings demonstrate a degree of robustness and consistency in the prognostic ability of the newly defined signatures of macrophage polarization across diverse diseases and independent datasets.

### SMaRT genes are significantly enriched in the macrophage proteome

**Fig. 5:**
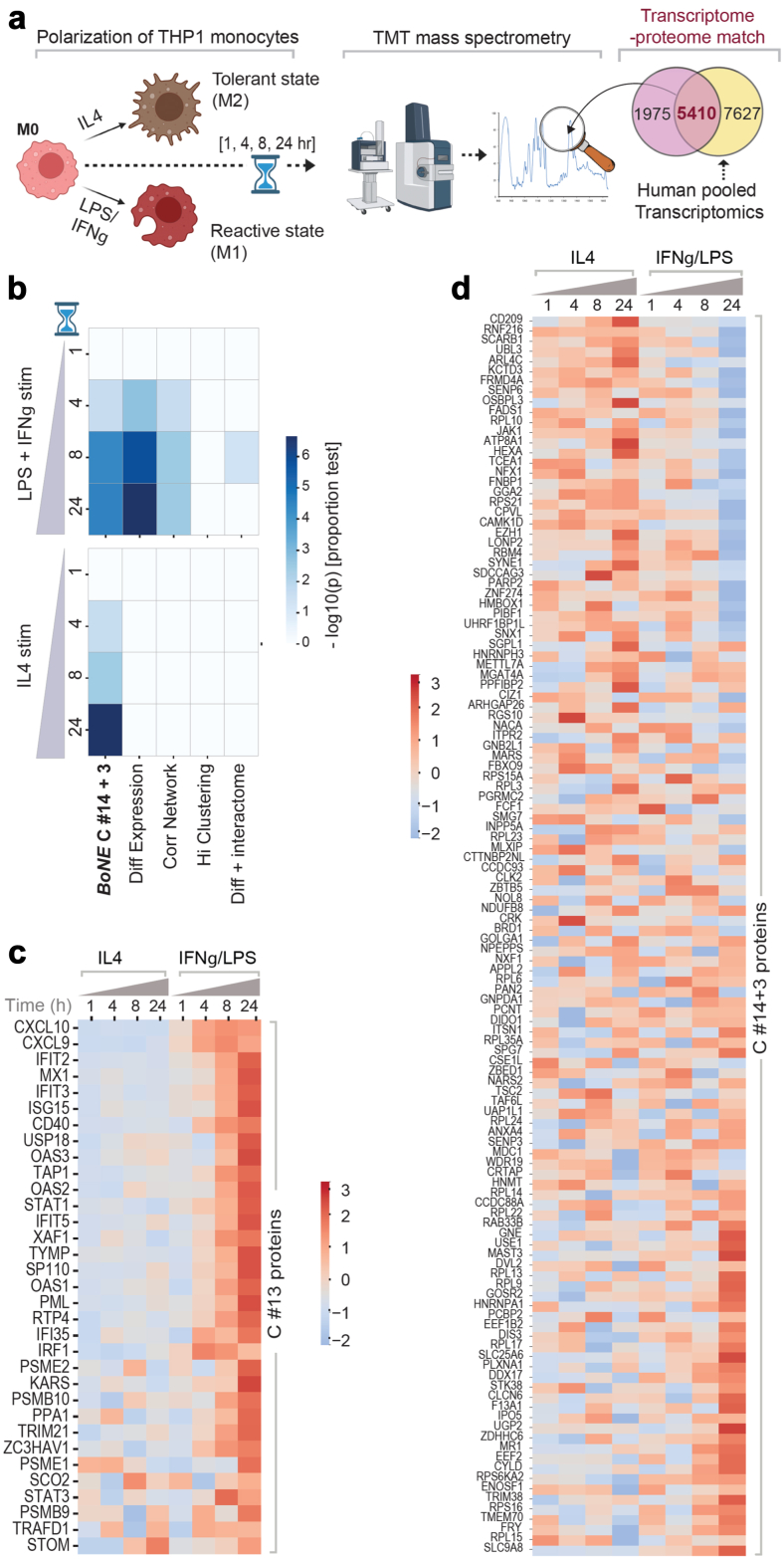
**SMaRT genes are differentially translated in polarized macrophages. a)** Overview of the experimental design. PMA-treated human THP-1 cell lines (M0) are polarized to M1 (with LPS and IFNγ) or M2 (with IL4), followed by multiplexed mass spectrometry at indicated time points. The fraction of the global macrophage transcriptome (from the pooled 197 macrophage datasets) that is represented in the global macrophage proteome is subsequently assessed for induction (or not) of proteins that are translated by various gene signatures. **b)** Selectivity of induction of proteins upon LPS and IFNγ (top) or IL4 (bottom) stimulation at various timepoints was assessed across different signatures using z-test of proportions and −log (10) p values are displayed as heatmaps. **c and d)** z normalized Log of intensities of proteins (Supplemental Information 3) translated at different time points by genes in C#13 (**c**) and C#14 + 3 (**d**) is displayed as heatmaps.

We used Tandem Mass Tag (TMT) proteomics datasets from THP1-derived macrophages (M0, PMA) that were polarized to M1-M2 states (see workflow Fig. 5a) and asked if the *BoNE*-derived gene clusters are translated to proteins. We found that the *BoNE*-derived *SMaRT* genes were induced significantly in the THP1 proteome (Supplemental Information 3). Consistent with our hypothesis that C#13 and path #14 → 3 carry independent information regarding “reactivity” and “tolerance”, we found that LPS and IFNγ-induced M1 polarization was associated with significant differential translation of genes in C#13 (Fig. 5b*-top*), whereas IL4-induced polarization was associated with significant differential translation of genes in C#14 and C#3 (Fig. 5b*-bottom*). Such differential protein translation continued to take place over 24 h (Fig. 5b).

Comparative analyses showed that while the “reactivity” signatures identified by two other conventional methodologies--Differential Expression and Correlation Network-- also reached significance; Fig. 5b*-top*), “tolerance” signatures derived by all other conventional approaches did not (Fig. 5b*-bottom*). Heatmaps show the dynamic and opposing nature of the proteins translated by the genes within the *BoNE*-derived gene signatures during polarization (Fig. 5c and d).

Findings demonstrate that the gene signatures of ‘reactivity’ and ‘tolerance’ identified here are significantly represented also in the translated proteome.

### Perturbation of SMaRT genes results in predictable outcomes

**Fig. 6:**
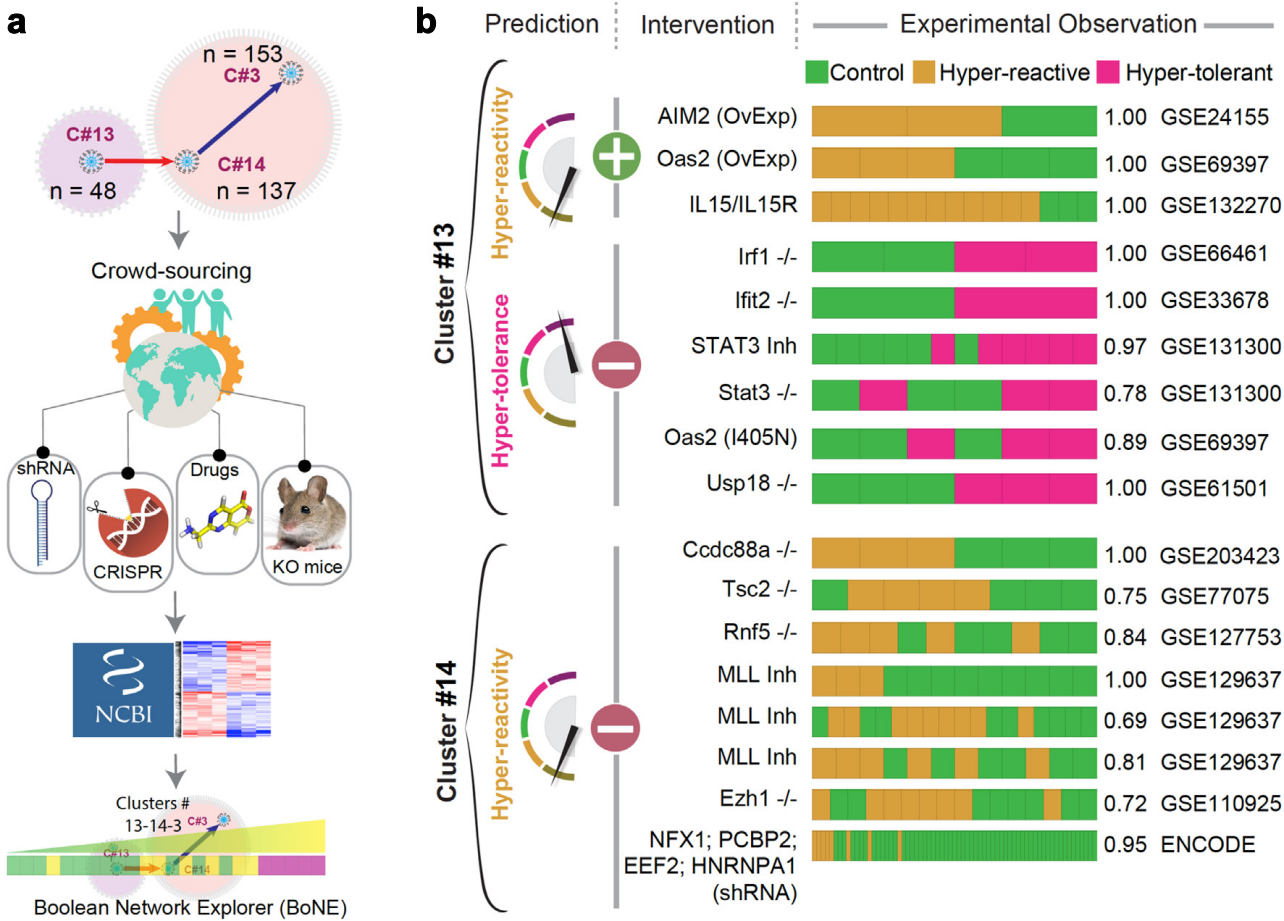
**Crowd-sourced assessment of the predictive potential of the *SMaRT* genes. a)** Overview of our workflow and approach for crowd-sourced validation. Publicly available transcriptomic datasets reporting the outcome of intervention studies (genetic or pharmacologic manipulations) on macrophages/monocytes targeting any of the 185 genes in C#13 and C#14 were analysed using the *BoNE* platform for macrophage states. **b)** Predicted impact of positive (+, either overexpression [OvExp] or agonist stimulations) or negative (−; genetic −/− models, shRNA, or chemical inhibitors) interventions and observed macrophage polarization states are shown. Performance is measured by computing ROC AUC for a logistic regression model. See Supplementary Table S3.

We next asked if network-rationalized interventions result in predictable outcomes upon perturbation, e.g., gene depletion (CRISPR, shRNA, KO mice) or overexpression, expression of functionally defective mutants, or chemical agonists/inhibitors. To this end, we carried out real-world crowdsourcing experiments on macrophage datasets in which interventions were conducted by different groups using diverse manipulations (Fig. 6a). In addition, we leveraged an existing asset within our own group, a previously validated myeloid specific *CCDC88A-KO*^82^ *(Ccdc88a*^*fl/fl*^*/LysM*^*Cre*^*)* model (CCDC88A belongs to C#14). Depletion or pharmacologic inhibition of any gene in C#13 was predicted to suppress reactivity and enhance tolerance, whereas overexpression or pharmacologic stimulation of the same should have an opposite impact, i.e., enhance reactivity and suppress tolerance. Similarly, depletion/inhibition of any gene in C#14 was predicted to enhance reactivity and suppress tolerance (Fig. 6b, *left* and Supplementary Table S3). The depletion of genes in C#3 is predicted to not have a robust impact on the network because of the Low => Low relationship with C#14.

We began with the ENCODE portal,^83^ a resource that was born out of the larger initiative called the ENCODE integrative analysis^84^; it is an encyclopedia of large, unbiased shRNA library screen on the human K562 chronic myeloid leukaemia cell line. This dataset contained 4 of the 137 genes in C#14 and none from C#13.^83^ In all 4 cases, the depletion of genes in C#14 resulted in the predicted outcome of enhanced reactivity and hypo tolerance (Fig. 6b, *right*). A systematic search of the NCBI GEO database also revealed 16 other independent datasets reporting the impact of interventions on genes in C#13 (9 datasets) and C#14 (7 datasets) (Supplementary Table S3). Regardless of the heterogeneous nature of the interventions and lab-to-lab variations in the type of cells/tissues used, predictions matched the observed outcomes in each instance. At least in one instance (i.e., STAT3), we could confirm the alignment of phenotypes between gene deletion and pharmacologic inhibition, implying that both approaches must have converged on the same biology. Because such alignment and/or convergence is seen in many instances,^85^ findings suggest that the current model can accurately guide outcome-driven pharmacologic interventions.

Together, these crowd-sourced studies rigorously and independently validate the definitions of macrophage polarization states; the fundamental nature of these definitions appear to remain relevant despite the thunderous heterogeneity of models and methods used by so many.

## Discussion

The lack of consensus on how to define macrophage activation has impeded progress in multiple ways; despite a panoply of existing descriptors, most remain contentious and/or confusing. AI-guided gene expression signatures presented here, *SMaRT*, offers a set of standardized definitions of macrophage polarization that encompasses four principles: (i) they are comprised of an unbiased collection of markers of macrophage activation that are represented in both the transcriptome and the proteome; (ii) they remain meaningful and relevant regardless of the source of macrophages (i.e., bone marrow, circulation, tissue-resident); (iii) they perform well across diverse activators, both in vitro and in vivo (i.e., recombinant ligands and cytokines, microbes, or multifactorial, as in the setting of complex disease states), and (iv) they provide a predictive framework that can be exploited for diagnostic purposes and for outcome-rationalized therapeutic interventions. These principles unify experimental standards for diverse experimental scenarios and interpretations across diverse tissues and diseases.

Finally, these *SMaRT* genes provide a common framework for macrophage activation nomenclature, which should enable laboratories to detect and report a given immunophenotype of macrophage in a standardized way. Standardization is expected to spur the development of robust strategies to address the multitude of macrophage-related disorders. It also serves as a starting point for the development of new diagnostics and immunomodulatory therapies.

## Contributors

Conceptualization: D.S, P.G.

Methodology: D.S, S.S, D.V., S.T., D.D.

Investigation: D.S, S.S, P.G.

Visualization: D.S, P.G, S.S, G.D.K, D.V.

Funding acquisition: D.S, S.D, P.G.

Project administration: D.S, P.G.

Supervision: D.S, P.G.

Writing—original draft: D.S, P.G.

Writing—review & editing: D.S, P.G, S.D, G.D.K, S.S.

D.S, and P.G have accessed and verified the underlying data. All authors read and approved the final version of the manuscript.

## Data sharing statement

All data are available in the main text or the supplementary materials. A website (http://hegemon.ucsd.edu/SMaRT/) of the macrophage network is built to support interactive query. The codes are available in SMaRT directory at https://github.com/sahoo00/BoNE.

## Declaration of interests

The authors declare that they have no financial conflict of interests for this study.
