# Supplementary material for: Machine learning identifies signatures of macrophage reactivity and tolerance that predict disease outcomes
Source: eBioMedicine. 2023 Jul 27;94:104719. doi: 10.1016/j.ebiom.2023.104719 (PMC10388732; doi:10.1016/j.ebiom.2023.104719)
Supplement: Supplementary Figs. S1–S9 and Tables S1–S3 [file mmc5.docx]

**Supplementary Materials**

**Machine Learning Identifies Signatures of Macrophage Reactivity and Tolerance that Predict Disease Outcomes**

**Authors:** Pradipta Ghosh^1-3^*^†^, Saptarshi Sinha^1,4^, Gajanan D. Katkar^1^, Daniella Vo^4^, Sahar Taheri^4^, Dharanidhar Dang^4^, Soumita Das^3,5,€^ and Debashis Sahoo ^3,4,6^*^†^

**Affiliations:**

^1^Department of Cellular and Molecular Medicine, University of California San Diego.

^2^Department of Medicine, University of California San Diego.

^3^Moores Cancer Center, University of California San Diego.

^4^Department of Pediatrics, University of California San Diego.

^5^Department of Pathology, University of California San Diego.

^6^Department of Computer Science and Engineering, Jacob’s School of Engineering, University of California San Diego.

^€^**Current Address**: Department of Biomedical and Nutritional Science, University of Massachusetts-Lowell, MA, USA.

**One Sentence Summary:** *S*ignatures of *ma*crophage *r*eactivity and *t*olerance (*SMaRT*) predict disease outcomes

*Contributed Equally

^†^**Corresponding authors:**

**Debashis Sahoo, Ph.D.;** Associate Professor, Department of Pediatrics, University of California San Diego; 9500 Gilman Drive, MC 0703, Leichtag Building 132; La Jolla, CA 92093-0831. **Phone:** 858-246-1803: **Fax:** 858-246-0019: **Email:** [dsahoo@ucsd.edu](mailto:dsahoo@ucsd.edu)

**Pradipta Ghosh, M.D.;** Professor, Departments of Medicine and Cell and Molecular Medicine, University of California San Diego; 9500 Gilman Drive (MC 0651), George E. Palade Bldg, Rm 232; La Jolla, CA 92093. **Phone**: 858-822-7633: **Email:** [prghosh@ucsd.edu](https://hsmail.ucsd.edu/owa/redir.aspx?C=TdXORuJOJYsSaKSg42oiljeLcKpf8UvqUqysJDu31kkqChk7oErWCA..&URL=mailto%3aprghosh%40ucsd.edu)

**This PDF file includes:**

Figure S1 to S9

Table S1 to S3

**Additional Data provided in separate files:**

**Supplemental Information 1**: Excel datasheet with an inventory of all publicly available gene expression datasets analyzed in this work.

**Supplemental Information 2**: List of 20 publicly available macrophage polarization and disease associated gene signatures. Clusters 13, 14, 3 list of genes are ranked based on equivalences within a cluster and the differential expression between M1 and M2.

**Supplemental Information 3**: Excel datasheet with list of proteins translated by genes in clusters #13 and 14+3 at various time points after ligand stimulation of THP1 cells.

**Supplemental Information 4**: Excel datasheet with ranked list of genes based on equivalences within a cluster and Reactome pathway analysis in the macrophage clustered Boolean implication network.

**Supplementary Figure Legends**


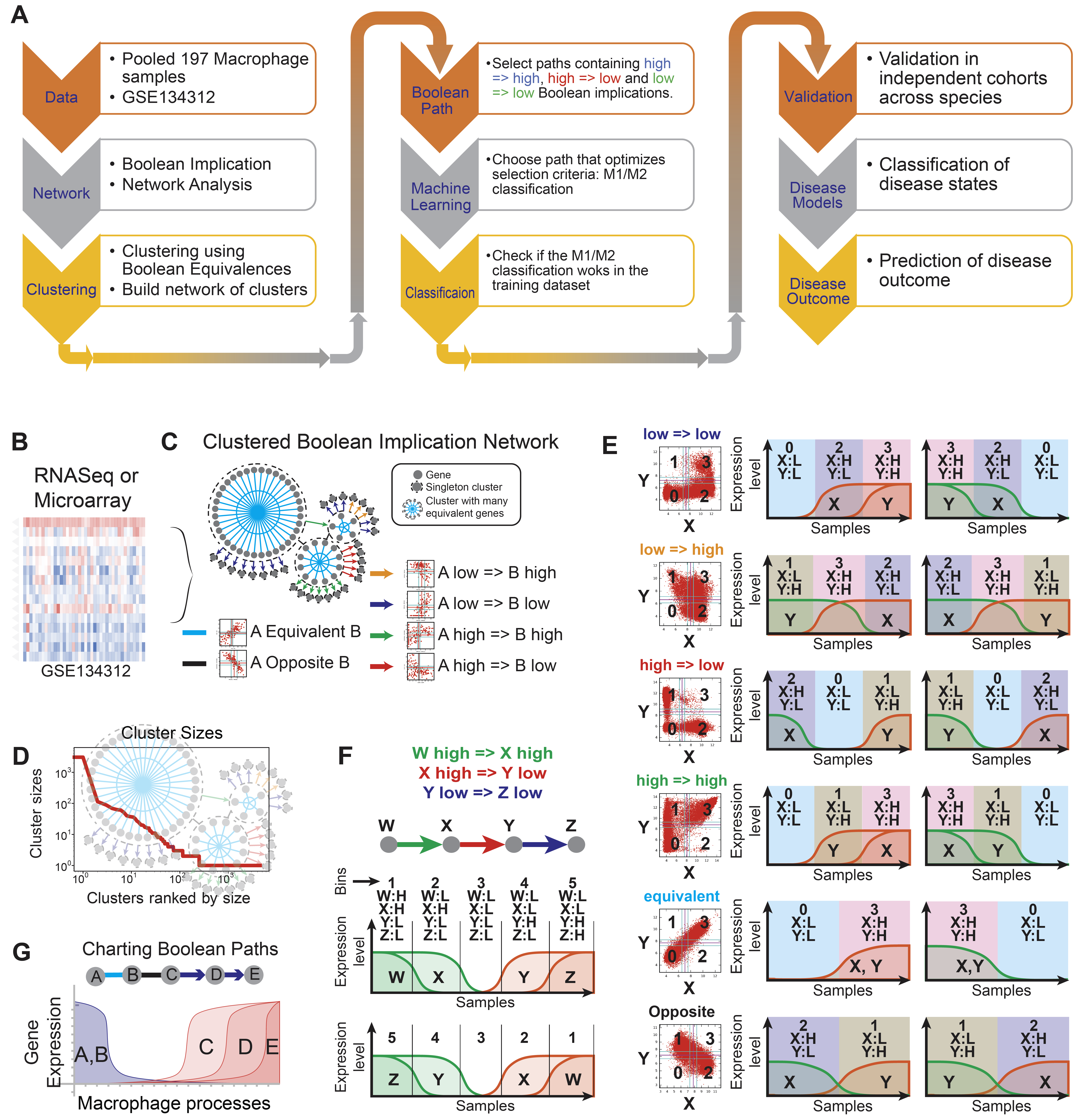


**Figure S1: Boolean Network Explorer (BoNE): A tool for clustering and visualization of the Boolean implication network**. **(A)** Overview of the computational steps used in *BoNE*. **(B)** *BoNE* was applied to analyze macrophage datasets to develop a model of polarization. GSE134312 is used to build the Boolean implication network. **(C)** BooleanNet algorithm is applied to identify Boolean implication relationships. The *BoNE* uses Boolean equivalent relationships to cluster genes and identify relationships between clusters. **(D)** A graphical display of cluster size analysis shows a linear trend in log-log scatterplots between clusters sorted by size and the number of clusters of any particular size. **(E)** Sample ordering based on single Boolean Implication relationships. **(F)** Sample ordering based on a sequence of high => high, high => low, low => low Boolean relationships. **(G)** Similar to panel F, four Boolean implication relationships ‘A equivalent to B’, ‘B opposite C’, ‘C low => D low’ and ‘D low => E’ low constitute a Boolean path that can be used to develop a computational model of macrophage polarization. A suitable path is selected by using machine learning that optimizes the strength of M1/M2 classification.


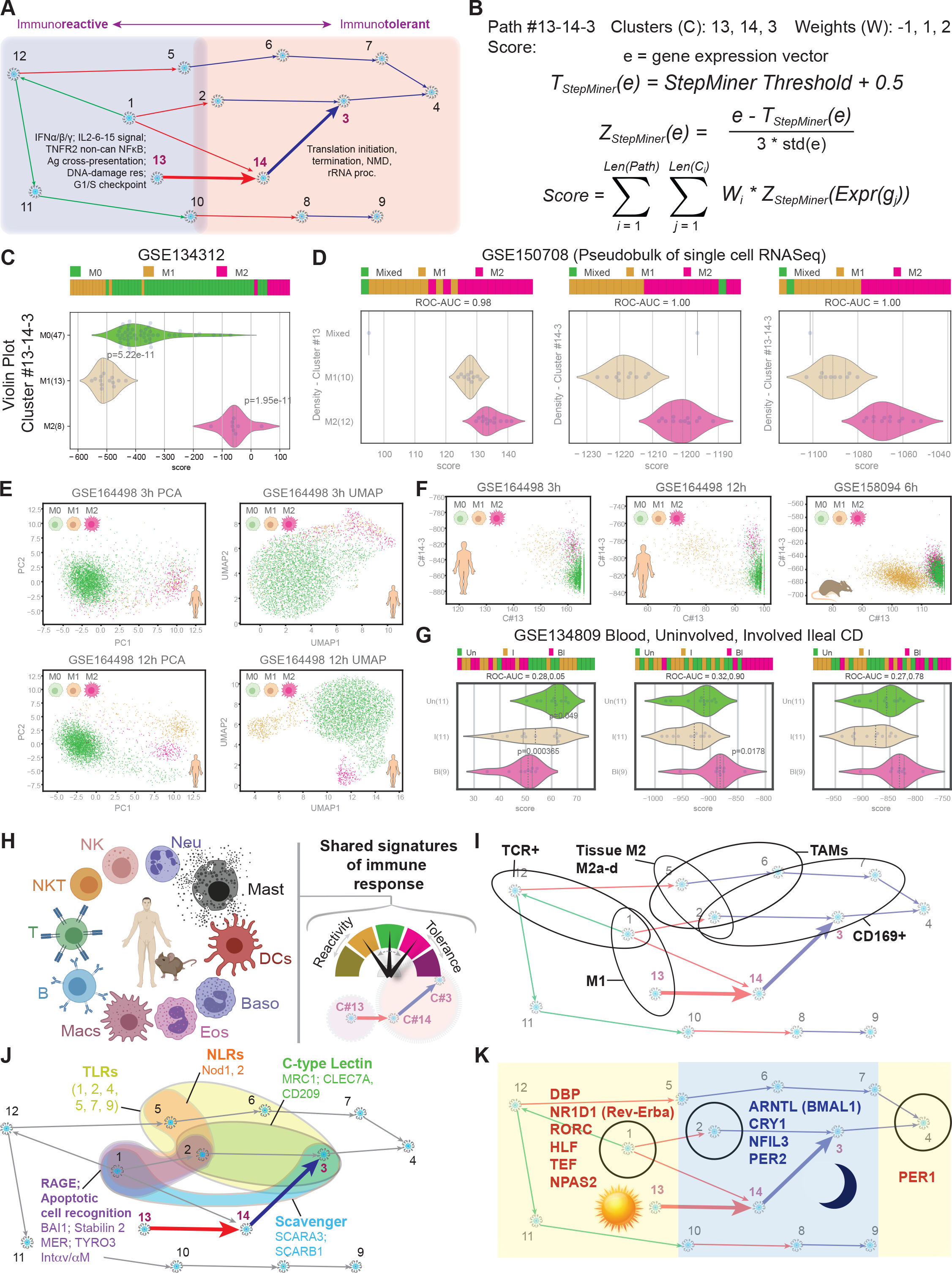


**Figure S2: Computational model of macrophage polarization: (A)** *BoNE* uses a Boolean implication network on macrophage dataset to build a computational model of macrophage polarization. Selected clusters by size were connected by high => high (green arrow), high => low (red arrows) and low => low (blue arrows) Boolean implication relationships. Reactome analysis of each cluster showed the biological processes in which the genes are involved.

**(B-C)** A path (#13-14-3) was selected in the network that is used to test M1/M2 states classification based on a score computed by using linear combination of normalized gene expression values (composite score; gj for j = 1 to len(Ci) is the list of genes in cluster Ci). This process is demonstrated by using a path #13-14-3 on GSE134312. P-values are derived from Welch's two-sided unpaired unequal variance Two Sample t-test between control and experimental samples.

(**D**) Test of M1 and M2 classification using cluster #13, path #14-3, and path #13-14-3 using artificial mixture of M1/M2 macrophages in single cell RNASeq dataset GSE150708. Mixed sample was created by combining 4000 lung cells, 4000 M1 and 4000 M2 macrophages such that final pseudo-bulk sample contains 33% M1 and 33% M2 macrophages. ROC-AUC values of M1 vs M2 classification are shown below each bar plot. The rest of the samples contain 10% of different combinations of M1 and M2 cells and 90% lung cells.

(**E**) PCA and UMAP analysis of human single cell dataset GSE164498 for 3h and 12h macrophage polarization protocols.

(**F**) Scatterplots of C#13 and path #14-3 composite scores in single cell RNASeq data GSE164498 (3h and 12h, human) and GSE158094 (6h, mouse).

(**G**) Bar and violin plots of pseudo-bulk analysis of computationally isolated macrophages (TYROBP > 2 and FCER1G > 2) from uninvolved and involved ileal biopsies and blood samples from Crohn’s disease patients.

(**H**) The schematic summarizes BoNE models (C#13, #14-3, #13-14-3) predicts activation states in diverse immune cell types.

(**I**) Known macrophage subtypes, as defined by marker genes, are projected on the Boolean map of macrophage processes. (**J**) The distribution of pattern recognition receptors (PRRs) [see **Table S2**] within various gene clusters of the Boolean map of macrophage processes is displayed.

(**K**) The positions of key circadian genes that are present in the network are shown on the Boolean map of macrophage processes. See also **Fig S3-4**.


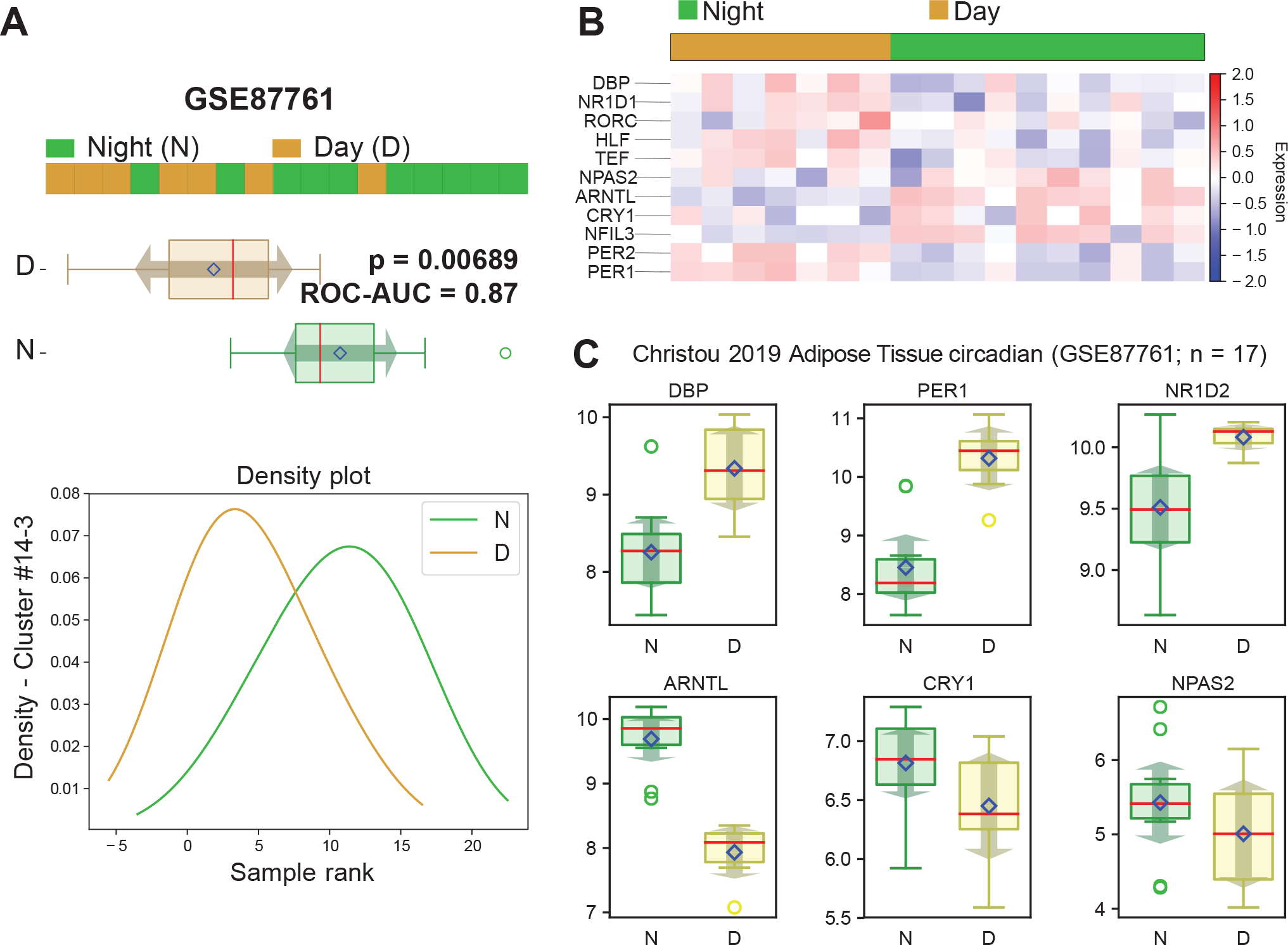


**Figure S3: Macrophages are reactive during the day and tolerant during the night.** **(A)**. Classification of macrophage polarization stages based on Boolean path #14-3 in GSE87761 (selected 17 samples from 33 using circadian phase as described below). This dataset contains tissue samples from subcutaneous adipose tissue that were taken at regular intervals under the highly controlled conditions of a strict sleep/wake and meal schedule. Circadian phases from -4 to 6 relative to dim light melatonin onset (DLMO) is considered night (N) and from 6 to 12 is considered day (D). A barplot (top), a boxplot (middle) and a density plot (bottom) are shown to demonstrate the ordering of samples using *BoNE* score based on path #14-3. The density plot is based on the rank of the score whereas barplot and boxplot are based on the raw *BoNE* score (path #14-3). The results show that night (N) is more tolerant compared to daytime (D). P-values are derived from Welch's two-sided unpaired unequal variance Two Sample t-test between control and experimental samples. ROC-AUC values of N vs D classification are shown below the bar plot. **(B)** Expression of important circadian genes found in the macrophage network in D and N samples is displayed as heatmap. **(C)** Boxplots displaying the levels of expression of circadian genes found in the macrophage network.

**Figure S4: Normalization of gene expression based on circadian rhythm:** **(A)** Overview of the normalization process. Normalization adjusts the *BoNE* score based on a clock gene (DBP, ARNTL, etc.). **(B)** Computation of original BoNE score based on a Boolean path. Example path #14-3 is used to demonstrate the score computation. StepMiner threshold + 0.5 is used to select the highest expression values. The expression values are scaled by using this threshold and the standard deviation. The *BoNE* score is computed by using a weighted linear combination of the scaled expression values. **(C)** Average values for each sample type are shown as colored diamonds. Maximum and minimum values are represented by bounding boxes. The *BoNE* score was adjusted based on maximum, minimum and average expressions of a clock gene in each sample type. The clock gene expression values were adjusted based on the *BoNE* score. **(D)** Linear regression was performed between the adjusted *BoNE* score and adjusted clock gene expression. Final normalized *BoNE* score was computed by subtracting the predicted trend. The final normalized score was used to rank the samples and visualized by using a bar+violin+swarm plot along with heatmap of sample types.

**
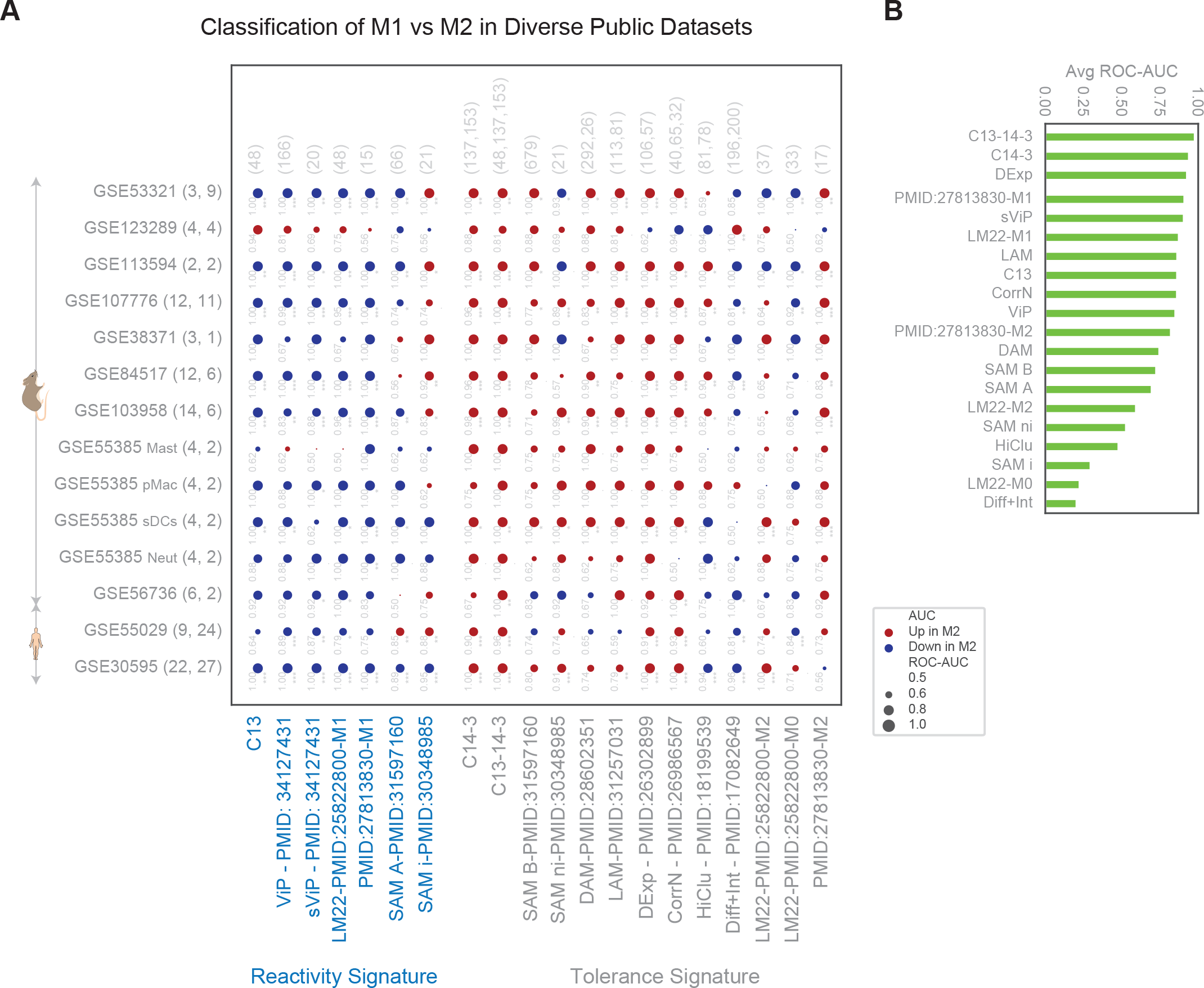
**

**Figure S5: Comparative analysis of Macrophage signatures.** **(A)**. Bubble plots of ROC-AUC values (radii of circles are based on the ROC-AUC, values are in gray) and the direction of gene regulation (Up in M2, red; Down in M2, blue) in 14 publicly available datasets (number of M1 and M2 samples are next to the GSE#, 2 human and 12 mouse; Neutrophil, Splenic DCs, Peritoneal Mac, and Mast cells included) using 20 gene signatures (composite score): proposed C#13-14-3, published macrophage polarization and disease-associated macrophage gene signatures (see **Supplemental Information 2**). Numbers on top of bubble plots indicate the number (n) of genes in each signature. P values based on Welch’s two sample unpaired two-sided t-test is represented using following codes: ‘.’ p≤0.1; *p ≤ 0.05; **p ≤ 0.01; ***p ≤ 0.001. (**B)** The gene signatures are ranked based on average of the ROC-AUC values across 14 datasets. For C#13, ViP, sViP, LM22-M1, PMID:27813830-M1, SAM A, Sam i the ROC-AUC values are flipped (1 - ROC-AUC) because they are upregulated in M1.

DExp, macrophage signature based on differential expression analysis (1); CorrN, correlation network analysis (2); HiClu, Hierarchical Clustering (3); Diff+Int, Differential Expression Analysis combined with interactome analysis (4); ViP, viral pandemic signature (5); sVip, severe Viral pandemic signature (5); LM22, 22 functionally defined human hematopoietic subsets signature (6); PMID:27813830, functionally validated macrophage regulators (7); LAMs, lipid-associated macrophages in atherosclerosis (8); DAMs, disease-associated microglia in neurodegenerative disorders (9); SAMs, scar-associated macrophages in liver fibrosis (10-12).


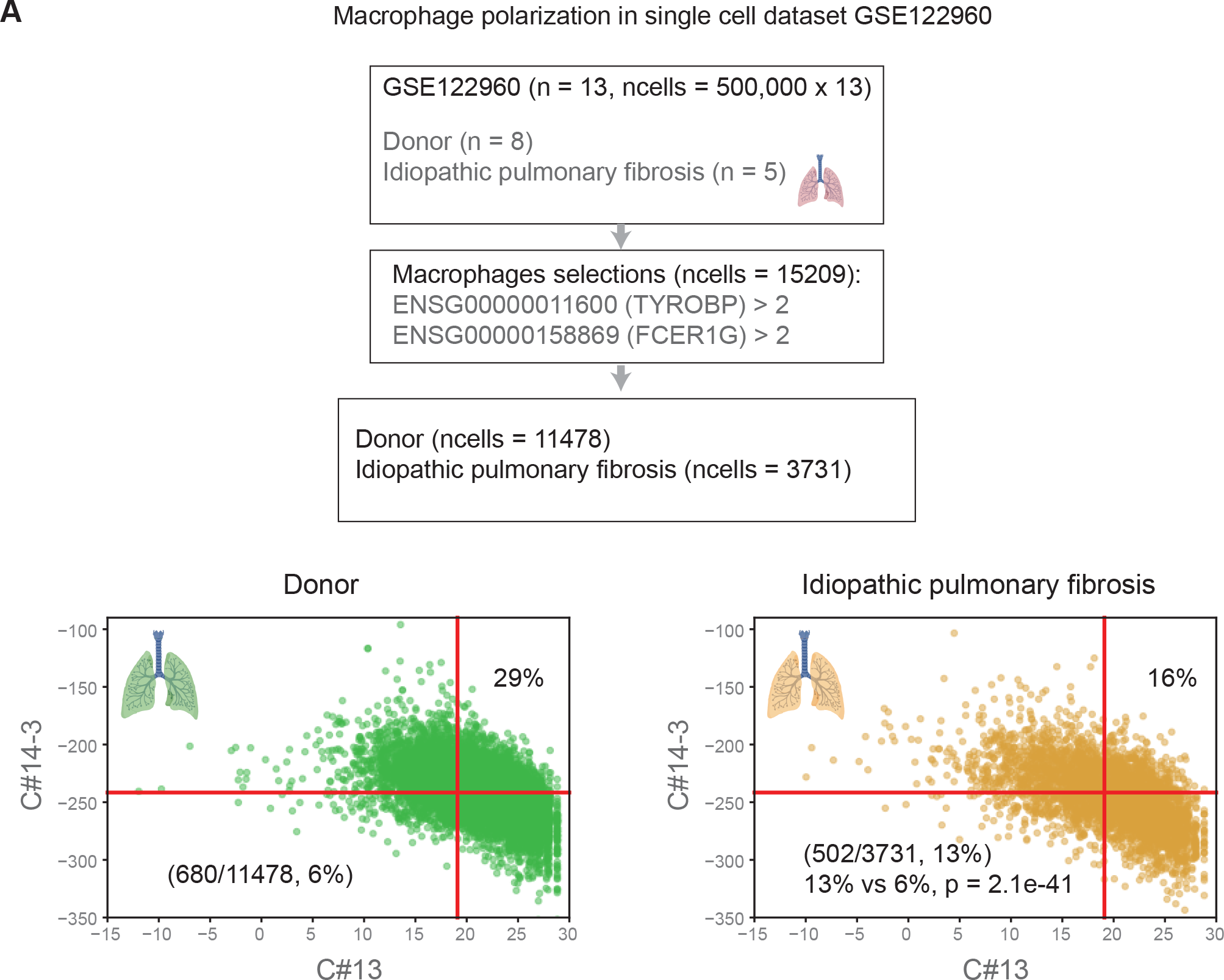


**Figure S6: Macrophage polarization analysis in single cell RNASeq dataset GSE122960.**

Macrophage polarization states were analyzed based on Cluster #13 and Boolean path #14-3 in single cell RNASeq dataset GSE122960 (n = 13, Donor: 8, Idiopathic pulmonary fibrosis: 5). 500,000 cells were selected from each sample for analysis. Macrophages (ncells = 15,209) were extracted by using raw count threshold (> 2) on ENSG00000011600 (TYROBP) and ENSG00000158869 (FCER1G) based on previous observation(13). Composite scores for C#13 and C#14-3 were calculated like the bulk RNASeq approach on the log2(CPM+1) values for single cell. StepMiner is used to compute thresholds (red lines) on the composite scores of C#13 and C#14-3. The bottom left quadrant is used to identify the percentage of “reactive” macrophages and top-right quadrant is used for the “tolerant” macrophages in each disease conditions. The composite scores are visualized in scatterplots (C#13 vs C#14-3) for each disease conditions. Two proportions two-tailed z-test is performed to test the significance of the “reactive” macrophages for each disease conditions compared to the donor control group.


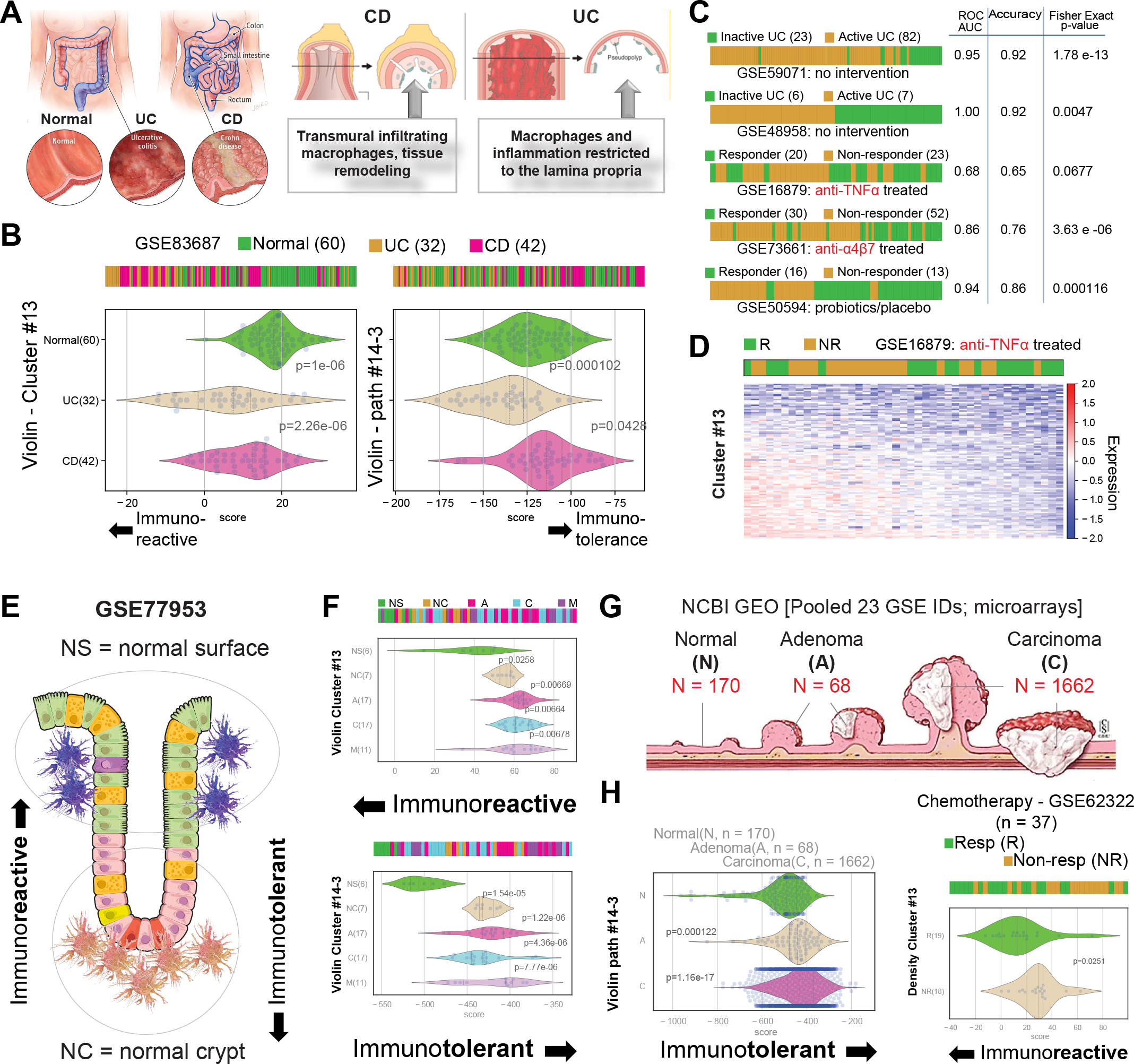


**Figure S7: Formal definitions of “reactivity” and “tolerance” identify the physiologic diversity of macrophages in the normal gut and the pathogenic responses in IBD and colorectal cancers (CRC).** (**A)** Normal, ulcerative colitis (UC) and Crohn’s disease (CD) samples were analyzed based on cluster #13 and path #14-3 gene signatures, which revealed distinct disease states. (**B**) Analysis of an IBD RNA Seq dataset (GSE83687, n = 134, 60 N, 32 UC, 42 CD) revealed hyper-reactive macrophage states in both UC and CD, but hyper-tolerant macrophage state exclusively in CD. P-values are derived from Welch's two-sided unpaired unequal variance Two Sample t-test between control and disease samples. **(C)** Levels of expression of genes in cluster #13 can distinguish responders *vs*. non-responders to treatment with: anti-α4β7 (GSE73661, n=82, 30 R, 52 NR); anti-TNFα (GSE16879, n=24, 8 R, 16 NR); standard therapies comprised of steroids, mesalamine, etc. (GSE59071, n=97, 23 R, 74 NR; and GSE48958, n=13, 6 R, 7 NR); placebo/probiotic (GSE50594, n=29, 16 R, 13 NR). Strength of classification is measured using ROC-AUC. Prediction strength using StepMiner threshold is measured using Accuracy, and Fisher Exact P-value. **(E)** Schematic summarizing the finding that the macrophages near the surface of normal crypt (NS) are more reactive than those near the base of normal crypt (NC). **(F)** Bar and violin plots show that Cluster 13 (top) and path 14-3 (bottom) gene signatures are differentially expressed in NS vs NC in laser-dissected tissues from the top and bottom of normal colon crypts and colon adenomas and CRCs (GSE77953, n=58, 6 NS, normal surface; 7 NC, normal crypt; 17 A, adenomas; 17 C, primary CRC; 11 M, metastatic CRC). (**G-H**) Cluster 13 and path 14-3 gene signatures analyzed on a pooled dataset (NCBI GEO dataset, 170 N, normal; 68 A, adenoma; 1662 C, Carcinoma) reveal a distinct progressive onset of tolerance in adenomas and CRCs compared to the normal colon (*left*) and that responders (R) to chemotherapy show higher reactivity than non-responders (NR) (GSE62322, n=37, 19 R, 18 NR) (*right*).

**Figure S8: Formal definitions of macrophage “reactivity” (R) and “tolerance” (T) identify pathologic states in diverse diseases.**

(**A**) General approach used towards the analyses in **Figure 4, S8 and S9**. R denotes reactivity and ‘T’ denotes tolerance; the arrows show the direction of progressive reactivity and/or tolerance based on the levels of expression of gene signatures. P-values are derived from Welch's two-sided unpaired unequal variance Two Sample t-test between control and disease samples.

(**B**) Summary of the two major types of arthritis-- rheumatoid arthritis (RA) involves swelling of synovium that may follow joint erosion, whereas osteoarthritis (OA) involves loss of cartilage between the joints.

(**C**) Bar and violin plots display the analyses on three publicly available arthritis cohorts (GSE55235, GSE55457 and GSE55584; n = 79; 20 HC, 33 RA, 26 OA), which revealed that macrophages are hyper-reactive in RA whereas they are hypo-reactive in OA. However, with respect to tolerance, they are both similar to healthy controls (HC).

(**D**) Multiple insults that trigger inflammation in the liver can cause progression to fibrosis (scarring), cirrhosis or liver cancer.

(**E-G**) Bar and violin plots display the analyses on publicly available liver datasets from non-alcoholic fatty liver disease (**E**; GSE89632, n=63; 20 FL, fatty liver; 19 SH, steatohepatitis; 24 HC, healthy controls), pooled cohorts of patients with alcoholic liver disease (**F**; GSE94417, GSE94397, GSE94399, n = 195, 109 C, control; 13 AH, alcoholic hepatitis; 6 AFL, alcoholic fatty liver; 67 AC, alcoholic cirrhosis) and viral liver disease (**G**; GSE70779, n=18; 9 Pre- and 9 Post-treatment with direct-acting anti-viral [post-DAA]).

(**H**) Among patients with Child-Pugh A cirrhosis, analysis of a prospective study (GSE15654, n = 216) showed that a higher reactive state (i.e., high expression of genes in cluster #13 *and* low expression of genes in #14-3) is unfavorable and is associated with a greater progression in Child-Pugh scores during follow-up.

(**I**) Reactive state in subcutaneous and visceral fat is associated with liver fibrosis (F) as compared to NASH+NAFLD (N) (GSE58979, n = 53, 10 F, 43 N).

(**J**) Schematic (*left*) summarizing the three major causes of the inflammation of the lung: smoking, asthma and chronic obstructive pulmonary disease (COPD); the latter is characterized by irreversible and progressive lung damage. Analysis (*right*) of two publicly available lung datasets (GSE2125, GSE13896, n = 115, 39 NS, non-smoker; 49 S, smoker; 15 A, asthma; 12, C, COPD) revealed that smoking and COPD are both hypo-reactive states (top) and that COPD is characterized by also hyper-tolerance (bottom).

(**K**) Schematic (*left*) summarizing the three major age groups included to study the impact of aging on macrophage processes. Analysis of a publicly available dataset (*right*) from peripheral monocytes from three independent donors (GSE60216, n=9, 3 N, neonate; 3 A, adult; and 3 O, old adult) unstimulated across 3 time-points.

(**L**) Schematic (*left*) shows the two different types of cardiomyopathies leading to heart failure, ischemic and non-ischemic. Analysis (*right*) of publicly available dataset from PBMCs from a cohort of heart failure patients (GSE104423; n = 25; 11 ICM, ischemic cardiomyopathy; 14 NICM, non-ischemic cardiomyopathy prior to undergoing mechanical circulatory support) and hearts of mice after experimental myocardial ischemia (GSE127244; n=16 at 0 and n=8 at 24 h) showed that ICM is associated with hyperreactivity and hypertolerance.

(**M**) Schematic showing the various neurodegenerative diseases and the regions of the brain that they involve.

(**N**) Analysis of multiple publicly available datasets representing a variety of neurodegenerative diseases. *From left to right*: AD, Alzheimer’s; HAND, HIV-associated neurodegenerative disease; FTD, frontotemporal dementia; DS, Downs Syndrome; CJD, Creutzfeld-Jakob Disease. In all conditions analyzed, diseased brains showed hyperreactivity as well as hypertolerance.

**Figure S9: Formal definitions of macrophage “reactivity” and “tolerance” identify pathologic states in sepsis, metabolic diseases, and cancers.**

(**A-D**) Macrophage polarization states based on Cluster #13 and Boolean path #14-3 in sepsis (**A)** (*Left*) GSE63042; 106 sepsis [S] and 23 systemic inflammatory response syndrome [SIRS]; (*Right*) GSE110487; 20 responders [R] and 14 non-responders [NR]). (**B**) GSE22309; 40 insulin-sensitive [IS], 40 insulin-resistant [IR] and 30 Diabetic [D]). (**C**) GSE98895; 20 control [C] and 20 metabolic syndrome [MetS]) and in sleep disorders (**D**). (**C**) For metabolic syndrome, analysis is shown before and after normalization to DBP, a gene that controls circadian rhythm. (**D**) For sleep disorders study, two datasets (GSE80612, GSE98582) were normalized to ARNTL, a gene that controls circadian rhythm (analyses are shown before and after normalization) and the other two datasets (GSE9444, GSE122541) were analyzed without any normalization.

(**E**) PBMC or lung tissue samples from humans infected with various respiratory viral infections and pandemics were classified based on either the levels of expression of genes in cluster #13 alone (*left*), clusters #14 and 3 (*middle*), or the path #13-14-3 (*right*). Numbers on the right side denote ROC-AUC values for classification accuracy compared to uninfected controls.

(**F**) Macrophage polarization based on Boolean path #13-14-3 is associated with outcome in several cancers, except in pancreatic cancer, where cluster #13 is prognostic.  P-values in the violin plots are derived from Welch's two-sided unpaired unequal variance Two Sample t-test between control and disease samples. P-values in the Kaplan-Meier plots are based on two-sided log-rank test.

**Table S1: M0, M1 and M2 annotation in GSE134312**

M0:

| GSM300403 | GSM300400 | GSM249415 | GSM343826 | GSM300398 | GSM249395 |
| --- | --- | --- | --- | --- | --- |
| GSM249417 | GSM343820 | GSM249389 | GSM249371 | GSM343808 | GSM249375 |
| GSM249367 | GSM343802 | GSM343810 | GSM343828 | GSM249409 | GSM360182 |
| GSM249407 | GSM249381 | GSM360186 | GSM343814 | GSM300402 | GSM249403 |
| GSM249385 | GSM360139 | GSM343812 | GSM300405 | GSM343806 | GSM300406 |
| GSM213500 | GSM300404 | GSM249377 | GSM343816 | GSM343818 | GSM343804 |
| GSM360143 | GSM343824 | GSM343830 | GSM300401 | GSM343822 | GSM249399 |
| GSM249423 | GSM300399 | GSM115052 | GSM115053 | GSM115054 | |

M1:

| GSM300391 | GSM300393 | GSM300396 | GSM213511 | GSM300390 | GSM300389 |
| --- | --- | --- | --- | --- | --- |
| GSM300392 | GSM300394 | GSM300397 | GSM300395 | GSM115055 | GSM115056 |
| GSM115057 | |  |  |  |  |

M2:

| GSM183209 | GSM183196 | GSM183193 | GSM183165 | GSM183201 | GSM115058 |
| --- | --- | --- | --- | --- | --- |
| GSM115059 | GSM115060 | |  |  |  |

**Table S2: List of pattern recognition receptors on macrophages and their position within the Boolean network of macrophage processes.**

| **Receptors Family** | **Gene name** | **Ligands (Microbial/cells)** | **Microbes/binding partners and unique Function** | **Cluster Number** |
| --- | --- | --- | --- | --- |
| **TLR Family^1^** | TLR1 | Triacyl lipoprotein | Bacteria  Works in conjunction with TLR2 | Cluster 2 |
|  | TLR2 | Lipoproteins, Peptidoglycans,  Lipoteichoic acids | Bacteria, Virus | Cluster 2 and 5 |
|  | TLR3 | double stranded RNA | Virus | absent |
|  | TLR4 | LPS | Bacteria | Cluster 2 |
|  | TLR5 | Flagellin | Bacteria | Cluster 2, 3, 5 |
|  | TLR6 | Diacyl lipoprotein | Bacteria, Virus  Works in conjunction with TLR2 | absent |
|  | TLR7 | Single stranded RNA | Bacteria, Virus | Cluster 6 |
|  | TLR8 | Single stranded RNA | Bacteria, Virus | Cluster 2 |
|  | TLR9 | Unmethylated DNA with the CpG motif | Bacteria, Virus, protozoa | absent |
|  | TLR10 | Unknown | Unknown | absent |
|  | TLR11 | Profilin like molecule | Protozoa | absent |
|  | TLR12 | Profilin | Protozoa | absent |
|  | TLR13 | 23s ribosomal RNA | Bacteria | absent |
|  |  |  |  |  |
| **C-type Lectin Receptor (CLR) family^2^** | Type 1-DEC205/CD205 | Binds apoptotic and necrotic cells |  | absent |
|  | Type 1-Macrophage mannose receptor (MMR)/MRC1 | Mannose, Fucose, N-acetyl glucosamine or glucose | binds mannose on the surface of pathogenic viruses, bacteria, and fungi so that they can be neutralized by phagocytic engulfment. | Cluster2 |
|  | Type 2-Dectin 1/CLEC7A | Binds beta glucan of fungal cell wall | Phagocytose live yeast and zymosan from fungi | Cluster 2 |
|  | Type 2-Dectin 2/CLEC6A | Binding with Pneumocystis major surface glycoprotein/ glycoprotein A (Msg/gpA) |  | absent |
|  | Type 2-Mincle/CLEC4E | α-mannose, Cord factor | FcRγ-coupled CLR that bind to mycobacterial cord factor as well as certain fungal species | absent |
|  | Type 2-DC-Sign/CD209 | Binds mycobacteria- 4 known ligands-DnaK, 60 kDa chaperonin-1 (Cpn60.1), GAPDH and lipoprotein lprG. (PMID: 21203928) |  | Cluster 3 |
|  | Type 2-DNGR1/CLEC9A | Lectin |  |  |
|  | MBL | Array of carbohydrates |  |  |
|  | MGL | N-acetylgalactosamine  (GalNAc) residues |  |  |
|  |  |  |  |  |
| **Scavenger Receptor^3^** | SRA  (SCARA1-5) | Lipid A of LPS; soluble LTA from Streptococcus pyogenes | Phagocytic receptor helps in the non-opsonic uptake of bacteria | SCARA3 in cluster 1 |
|  | MARCO | Soluble LPS and LTA, CpG DNA, intact Gram-positive and Gram-  -negative bacteria | Host defense against S pneumonae infection in mouse model | absent |
|  | CD36 | Bacterial membrane, LPS |  | absent |
|  | SRB1/SCARB1 | Binds high density lipoprotein |  | Cluster 1 and 3 |
|  |  |  |  |  |
| **Nod like receptor (NLR)^4^** | Nod1 | Di-amino-pimelic Acid | Cytosolic sensor, activates host signaling by Rip2 | Cluster 1 |
|  | Nod2 | Muramyl dipeptide |  | Cluster 2 and 5 |
|  | Naip | Flagellin |  | absent |
|  | NLRC3 | Unknown |  | absent |
|  | NLRC4 | Flagellin |  | absent |
|  | NLRP1 | Muramyl dipeptide |  | absent |
|  |  |  |  |  |
| **Receptor for advanced glycation end-products (RAGE)^5^** | RAGE | Multi ligands: AGEs, HMGB1, S100s, beta sheet fibrils, DNA, RNA |  | Cluster 1 |
|  |  |  |  |  |
| **Apoptotic cell recognition by direct binding^6^** | BAI1 | Phosphatidyl Serine, bacterial LPS | GPCR- ELMO1, DOCK, Rac1 | Cluster 1 |
|  | TIM1 | Phosphatidyl Serine, hepatitis virus A | Signal via Fyn Kinase |  |
|  | TIM4 | Phosphatidyl Serine | Indirect via integrins | Absent |
|  | Stabilin 2 | Phosphatidyl Serine | ITIM | Cluster 1 |
|  | CD300 | Phosphatidyl Serine | Via GULP | Absent |
| **Apoptotic cell recognition by indirect binding^7^** | MER- type I receptor tyrosine kinase | Protein S (PROS1) |  | Cluster 1 |
|  | AXL- type I receptor tyrosine kinase | GAS6 |  | Absent |
|  | Tyro3- type I receptor tyrosine kinase | GAS6, PROS1 |  | Cluster 1 |
|  | SCARF | C1q |  | absent |
|  | Integrin aMb2 | C1q |  | Cluster 1 |
|  | Integrin avb3 | MFG-E8 | CRKII, DOCK180, Rac1 | Cluster 2 |
|  | Integrin avb5 | MFG-E8 | FAK, DOCK180, Rac1 | Cluster 2 |
|  | CD36 | oxidized low-density lipoprotein (oxLDL), anionic phospholipids, long-chain fatty acids, thrombospondin-1 (TSP1), fibrillar β-amyloid | Fyn, PYK2 | absent |

1-7 collected from Fox & Das, Book Chapter, 2015

^1^ PMID: 23284045; PMID: 12524386, PMID: 11905821; PMID: 20303872

^2^ PMID: 29497419; PMID: 32060644

^3^ PMID: 30318962

^4^ PMID: 20303872

^5^ PMID: 15488742

^6,7^ PMID: 26683144; PMID: 29400998

**Table S3: Datasets identified in NCBI GEO database in which the expression or function of targets in cluster #13 or #14 were experimentally manipulated in cells of the myeloid lineage.**

| Genes | GSEID | Model/approach | Species | Predicted outcome | Observed outcome |
| --- | --- | --- | --- | --- | --- |
| AIM2 | GSE24155 | Overexpression, HCT116 (colorectal tumour cell line) | Human | Hyper-reactivity | Hyper-reactive |
| Oas2 | GSE69397 | KO mice | Mouse | Hyper-reactivity | Hyper-reactive |
| IL15/IL15R | GSE132270 | Recombinant IL15 Monocytes | Human | Hyper-reactivity | Hyper-reactive |
| IRF1 | GSE66461 | KO mice | Mouse | Hyper-tolerence | Hyper-tolerant |
| IFIT2 | GSE33678 | KO mice | Mouse | Hyper-tolerence | Hyper-tolerant |
| STAT3 | GSE131300 | Inhibitor ruxolitinib, Primary human macrophage | Human | Hyper-tolerence | Hyper-tolerant |
| STAT3 | GSE131300 | KO mice, BMDM | Mouse | Hyper-tolerence | Hyper-tolerant |
| Oas2 (I405N) | GSE69397 | KO mice | Mouse | Hyper-tolerence | Hyper-tolerant |
| USP18 | [GSE61501](https://www.ncbi.nlm.nih.gov/geo/query/acc.cgi?acc=GSE61501) | KO mice, Microglia | Mouse | Hyper-tolerence | Hyper-tolerant |
| CCDC88A |  | KO mice, Peritoneal macrophage | Mouse | Hyper-reactivity | Hyper-reactive |
| TSC2 | [GSE77075](https://www.ncbi.nlm.nih.gov/geo/query/acc.cgi?acc=GSE77075) | KO mice, BMDM | Mouse | Hyper-reactivity | Hyper-reactive |
| RNF5 | [GSE127753](https://www.ncbi.nlm.nih.gov/geo/query/acc.cgi?acc=GSE127753) | KO mice | Mouse | Hyper-reactivity | Hyper-reactive |
| MLL | GSE129637 | NPM1c knock-in cells, inhibitor VTP-50469 | Mouse | Hyper-reactivity | Hyper-reactive |
| MLL | GSE129637 | AML cell lines carrying NPM1c mutations, inhibitor VTP-50469 | Human | Hyper-reactivity | Hyper-reactive |
| MLL | GSE129637 | inhibitor VTP-50469 |  | Hyper-reactivity | Hyper-reactive |
| EZH1 | [GSE110925](https://www.ncbi.nlm.nih.gov/geo/query/acc.cgi?acc=GSE110925) | KO mice | Mouse | Hyper-reactivity | Hyper-reactive |
| NFX1, PCBP2, EEF2, HNRNPA1 | ENCODE | ShRNA | Human | Hyper-reactivity | Hyper-reactive |

**References:**

1. Becker M, De Bastiani MA, Parisi MM, Guma FT, Markoski MM, Castro MA, et al. Integrated Transcriptomics Establish Macrophage Polarization Signatures and have Potential Applications for Clinical Health and Disease. Sci Rep. 2015;5:13351.

2. Bell LC, Pollara G, Pascoe M, Tomlinson GS, Lehloenya RJ, Roe J, et al. In Vivo Molecular Dissection of the Effects of HIV-1 in Active Tuberculosis. PLoS Pathog. 2016;12(3):e1005469.

3. Coates PJ, Rundle JK, Lorimore SA, Wright EG. Indirect macrophage responses to ionizing radiation: implications for genotype-dependent bystander signaling. Cancer Res. 2008;68(2):450-6.

4. Martinez FO, Gordon S, Locati M, Mantovani A. Transcriptional profiling of the human monocyte-to-macrophage differentiation and polarization: new molecules and patterns of gene expression. J Immunol. 2006;177(10):7303-11.

5. Sahoo D, Katkar GD, Khandelwal S, Behroozikhah M, Claire A, Castillo V, et al. AI-guided discovery of the invariant host response to viral pandemics. EBioMedicine. 2021;68:103390.

6. Newman AM, Liu CL, Green MR, Gentles AJ, Feng W, Xu Y, et al. Robust enumeration of cell subsets from tissue expression profiles. Nat Methods. 2015;12(5):453-7.

7. Murray PJ. Macrophage Polarization. Annu Rev Physiol. 2017;79:541-66.

8. Jaitin DA, Adlung L, Thaiss CA, Weiner A, Li B, Descamps H, et al. Lipid-Associated Macrophages Control Metabolic Homeostasis in a Trem2-Dependent Manner. Cell. 2019;178(3):686-98.e14.

9. Keren-Shaul H, Spinrad A, Weiner A, Matcovitch-Natan O, Dvir-Szternfeld R, Ulland TK, et al. A Unique Microglia Type Associated with Restricting Development of Alzheimer's Disease. Cell. 2017;169(7):1276-90.e17.

10. Duffield JS, Forbes SJ, Constandinou CM, Clay S, Partolina M, Vuthoori S, et al. Selective depletion of macrophages reveals distinct, opposing roles during liver injury and repair. J Clin Invest. 2005;115(1):56-65.

11. Ramachandran P, Dobie R, Wilson-Kanamori JR, Dora EF, Henderson BEP, Luu NT, et al. Resolving the fibrotic niche of human liver cirrhosis at single-cell level. Nature. 2019;575(7783):512-8.

12. MacParland SA, Liu JC, Ma XZ, Innes BT, Bartczak AM, Gage BK, et al. Single cell RNA sequencing of human liver reveals distinct intrahepatic macrophage populations. Nat Commun. 2018;9(1):4383.

13. Dang D, Taheri S, Das S, Ghosh P, Prince LS, Sahoo D. Computational Approach to Identifying Universal Macrophage Biomarkers. Front Physiol. 2020;11:275.
